# Analysis of Physical Processes in Confined Pores of Activated Carbons with Uniform Porosity

**DOI:** 10.3390/ma19010191

**Published:** 2026-01-04

**Authors:** Magdalena Blachnio, Malgorzata Zienkiewicz-Strzalka, Anna Derylo-Marczewska

**Affiliations:** Department of Physical Chemistry, Institute of Chemical Sciences, Maria Curie-Sklodowska University, Maria Curie-Sklodowska Square 3, 20-031 Lublin, Poland; malgorzata.zienkiewicz-strzalka@mail.umcs.pl

**Keywords:** mesoporous activated carbon, phase transition in confined pores, dye adsorption, methylene blue, Bismarck brown, reactive black, nanostructure

## Abstract

Mesoporous carbons based on silica hard templates were used to investigate physical processes in confined pores. Nitrogen adsorption, scanning electron microscopy, and scattered X-ray analyses revealed two classes of materials: carbons with moderate and highly developed mesoporosity. The pore structure was strongly dependent on pore expanders which proved essential for generating open, accessible architectures. All carbons exhibited a basic, graphitic surface (pH_PZC_ = 8.4–10.9), enriched in electron-donating oxygen functionalities. Differential scanning calorimetry studies of confined water showed that melting point depression follows the Gibbs–Thomson relationship, confirming the strong dependence of phase transitions on pore size and water–surface interactions. Adsorption experiments using methylene blue demonstrated that capacity is governed by surface area, pore volume, and pore size distribution. For carbon with the largest average pore size, adsorption of various dyes revealed that uptake decreases with increasing molecular size, whereas affinity depends strongly on electrostatic interactions. Kinetic studies indicated that carbons with larger mesopores exhibit the fastest adsorption, and that large, complex dye molecules undergo significant diffusion limitations. Overall, the results show that the interplay between pore structure, adsorbate size, and surface chemistry influences both the equilibrium uptake and adsorption kinetics in mesoporous carbon materials.

## 1. Introduction

Carbon adsorbents encompass a broad spectrum of materials, including activated carbons, carbon molecular sieves, activated carbon fibers, and carbon membranes [1,2,3,4,5]. These materials share several fundamental features such as a high carbon content (typically above 90%), a well-developed internal porosity, a large specific surface area, and a hydrophobic surface character [6,7]. Such characteristics enable them to selectively capture molecules from both gaseous and liquid phases [8]. In recent years, a distinct class of nanostructured carbon-based adsorbents has emerged, namely, porous ordered carbons synthesized through inorganic templating strategies [9,10]. These advanced nanomaterials integrate the intrinsic properties of carbon such as high surface area, electrical conductivity, and chemical stability with the structural ordering provided by inorganic matrices, most commonly mesoporous silicas (e.g., SBA-15, MCM-41) [11]. The inorganic template dictates the ordered pore architecture, which not only enhances the accessibility of the active surface but also significantly improves mass and ion transport. This structural control is particularly advantageous in electrochemical applications, where ion mobility and electrical conductivity are critical for performance [12,13]. The stable and directed pore architecture of ordered porous carbons enables the design of materials with highly specific functionalities such as creating specific reactors for chemical processes and reactions. Reactions in the pores of porous carbon materials involve the controlled interaction of the carbon’s surface or incorporated materials with reactants, where the pore structure’s size, distribution, and surface area dictate the reaction’s kinetics and selectivity. Moreover, the finite size of pores leads to rounding or broadening of phase transitions. Unlike in bulk systems, where the order parameter changes sharply at the transition, confined systems exhibit gradual variations in density, enthalpy, or structure over a range of conditions. As a result, they have been intensively investigated for a wide range of advanced applications, including electrodes in supercapacitors and rechargeable batteries, catalyst supports, sorbents for gas and liquid phase separation, membranes, biosensors, and hydrogen storage systems for energy-related technologies [14,15].

In the case of the pore reactor concept, the carbonaceous materials create micro- or nanoscale reaction environments within the interior of their pores or channels, where reactants diffuse and undergo targeted chemical transformations. The dynamics of exchange between the in-pore and ex-pore phases and the influence of factors such as carbon particle size and porosity degree determine to a large extent the processes conducted in internal pore space [16]. The structural confinement provided by the pores enhances reactant–catalyst interactions, facilitates selective reactions, and can improve reaction kinetics by reducing diffusion limitations [17]. Moreover, the porous framework itself can be functionalized or doped to introduce catalytic species, such as metal nanoparticles, metal oxides, or functional groups, or it can act as a support for pre-deposited catalysts [18]. Providing a confined reaction space and hosting active catalytic sites enables efficient utilization of reagents, higher selectivity, and enhanced stability under continuous reaction conditions. Consequently, ordered porous carbons are increasingly applied in flow-through reactors, micro- and nano-reactors, and heterogeneous catalysis, where they contribute to improved mass transport, optimized reactant accessibility, and controlled reaction environments, which are critical for advanced chemical synthesis, energy conversion, and environmental applications [19,20,21,22]. This issue is deepened by scientific research in the field of chemical processes that take place in the pores of porous activated carbons as simulation of the composition of mixtures reacting in the pores in comparison with gas-phase systems [23].

In addition to the chemical reactions, also the physical processes taking place in the confined pore systems are strongly dependent on their structural characteristics. The experimental measurements and molecular simulations of phase transitions, including melting and solidification of simple fluids (carbon tetrachloride and nitrobenzene) in porous media, were investigated in work [24]. Experimental and theoretical studies have shown that the freezing and melting points of fluids confined in nanoporous carbons are strongly dependent on pore size. Cuadrado-Collados et al. [25] demonstrated that water confined in the nanospace of activated carbon exhibits a pronounced depression of both freezing and melting temperatures relative to the bulk, with no detectable phase transition occurring in ultramicropores (<0.7 nm) under ambient pressure. Similarly, simulations and adsorption studies have revealed that capillary condensation and evaporation of fluids in carbon pores occur over shifted pressure ranges and exhibit pronounced hysteresis loops [26,27]. The magnitude of these shifts follows curvature-dependent relations such as the Gibbs–Thomson or Kelvin equations, in which the equilibrium pressure or temperature depends inversely on the effective pore radius [28,29]. In carbon materials, the combination of heterogeneous surface chemistry and complex pore connectivity further broadens or smooths these transitions, leading to gradual rather than sharp phase changes. These and many other works [26,30,31,32,33,34] suggest that when a fluid or mixture is confined within pores, its phase behavior differs significantly from that in the bulk state. The confinement, resulting from spatial restrictions and surface interactions, modifies thermodynamic equilibrium, alters the nature and position of phase transitions, and often gives rise to phenomena that have no analogues in macroscopic systems.

The aim of this work was to investigate the influence of pore structure and surface chemistry of mesoporous carbons obtained from silica hard templates on the course of physical processes such as the liquid–solid phase transition occurring within confined pore spaces. Furthermore, this study aimed to determine the relationships between the properties of dyes and mesoporous carbons, as well as their influence on the equilibrium and kinetics of adsorption process. The influence of several factors related to the textural, structural, and surface properties of adsorbents, molecular size, and physicochemical properties of adsorbates on adsorption processes was analyzed, with the overall effectiveness of this technique in mind in terms of optimal adsorption capacity and adsorption rate. A comprehensive analysis of physical processes in model porous systems allows for the identification of certain relationships between structure and properties, enabling the optimization of material properties for specific applications.

## 2. Materials and Methods

### 2.1. Chemicals and Materials

#### 2.1.1. Chemicals

Glucose, sulphuric acid H_2_SO_4_ (concentration 95%), potassium hydroxide (KOH), and methylene blue (MB) were purchased from Avantor Performance Materials Poland S.A. (Gliwice, Poland). Sodium chloride, sodium hydroxide, Bismarck brown (BB), and reactive black (RB) were purchased from Sigma Aldrich (Poznań, Poland).

#### 2.1.2. Synthesis of Carbon Materials

Carbon materials were synthesized by a hard-templating method via direct replication of a mesoporous silica matrix with an ordered structure. The detailed procedure for the preparation of MCF silica is described elsewhere [35]. In the first step, the silica templates were impregnated with an aqueous solution of glucose in the presence of sulfuric acid (VI) under vacuum conditions at 160 °C. The obtained silica–organic composites were subjected to carbonization in a nitrogen atmosphere at 700 °C for 4 h, leading to the formation of carbon replicas within the silica framework. Finally, the silica matrix was removed by chemical etching in a 1 M KOH solution prepared in a mixed solvent system of H_2_O/C_2_H_5_OH (50/50, *v*/*v*) at 100 °C for 1 h, yielding the mesoporous carbon materials.

### 2.2. Textural and Structural Characterization of Porous Carbon Materials

The porous structure of the carbon materials was characterized based on nitrogen adsorption–desorption measurements at 77 K over a relative pressure range from 0 to 950 mmHg using an ASAP 2020 analyzer (Micromeritics, Norcross, GA, USA). The specific surface area (S_BET_) was determined from the adsorption isotherm according to the standard Brunauer–Emmett–Teller (BET) method. The pore size distribution was derived from both adsorption and desorption branches of the isotherm using the Barrett–Joyner–Halenda (BJH) model for cylindrical pores with Faas correction. The total pore volume (V_t_) was estimated from the amount of nitrogen adsorbed at a relative pressure (p/p_0_) of 0.97, while the external surface area, S_ext_, and mesopore volume, V_p_, were determined by the α_s_ method using non-graphitized Carbon Black Cabot BP 280 as a standard [36]. Prior to analysis, the carbon sample was degassed at 100 °C under vacuum (1 mmHg) for 24 h in the analyzer’s degassing port.

The acid–base properties of the carbon surface were evaluated by potentiometric titration. A suspension containing 0.1 g of carbon in 30 mL of NaCl solution was acidified and placed in a thermostated vessel maintained at 25 °C. The suspension was titrated with a NaOH solution using an automatic burette (Dosimat 765, Metrohm, Herisau, Switzerland) connected to a pH meter (PHM240, Radiometer, Copenhagen, Denmark). Based on the recorded pH variations as a function of titrant volume, the surface charge density and the point of zero charge (pH_pzc_) of the activated carbon were determined.

The scanning electron microscopy (SEM) analysis was carried out using a Phenom™ ProX G6 device (Thermo Fisher Scientific Inc., Waltham, MA, USA) operated at 20 kV. Imaging of the hydrogel samples was conducted under vacuum conditions (1 Pa).

The surface chemistry of the carbon materials, including information on surface-active groups, elemental composition, and the electronic states of the constituent elements, was examined using X-ray photoelectron spectroscopy (XPS). The XPS measurements were performed on a Multi-Chamber UHV System (Prevac, 2009, Rogów, Poland) equipped with a hemispherical Scienta R4000 analyzer and a high-intensity monochromatic Al Kα source (MX-650, Scienta, Uppsala, Sweden).

SAXS measurements were performed on an Empyrean diffractometer (PANalytical, Almelo, The Netherlands) equipped with a Cu anode X-ray tube and a SAXS/WAXS sample stage operated in capillary mode. The instrument was powered by a 4 kW high-voltage generator set to 40 kV and 40 mA. The incident beam was conditioned using a W/Si graded elliptical mirror. Data were collected over a 2θ range from −0.1° to 4°, with a step size of 0.005° and a counting time of 1.76 s per step, yielding ~800 data points per scan. A 0.2 mm Cu beam attenuator was used for measurements near the primary beam. Scattered X-rays were detected with a PIXcel3D detector through a receiving slit of 0.05 mm active length. The scattering vector was defined as q = 4πsinθ/λ where θ is the scattering angle and λ = 1.5418Å is the Cu Kα radiation wavelength. Background scattering was determined from air measurements using an empty capillary holder and subtracted from the sample data.

### 2.3. Phase Transition Measurements in Confined Pores

Phase transition measurements of water confined within solid pores were carried out using differential scanning calorimetry (DSC) with an STA 449 Jupiter F1 instrument (Netzsch, Selb, Germany). Prior to analysis, each carbon sample was vacuum-dried at 100 °C for 24 h, immersed in distilled water at a 1:10 solid-to-liquid ratio, shaken in a New Brunswick incubator shaker at 25 °C for 48 h, filtered, and finally dried at room temperature for 2 h. Approximately 10 mg of the prepared sample was sealed in an alumina crucible. The DSC measurements were performed under a helium atmosphere within the temperature range of −100 °C to 500 °C, at a cooling rate of 20 °C min^−1^ and a heating rate of 10 °C min^−1^.

### 2.4. Dye Adsorption Isotherm and Kinetic Measurements

The adsorption of methylene blue (MB), Bismarck brown (BB), and reactive black (RB) dyes from aqueous solutions onto mesoporous carbon materials was carried out using a batch (static) method. A series of adsorption systems was prepared by adding 25 mL of the dye solution with a defined initial concentration to Erlenmeyer flasks containing approximately 0.05 g of the adsorbent. The adsorption experiments were conducted at 25 °C, with a stirring speed of 120 rpm, for 7 days in the case of MB and BB dyes, and 14 days for RB. After the equilibrium period, the solutions were filtered and analyzed spectrophotometrically using a UV–Vis spectrophotometer (Cary 4000, Varian Inc., Melbourne, VIC, Australia).

The kinetic studies of dye adsorption on the carbon materials were performed using a continuous spectral recording method. The experiments were initiated by introducing 50 mL of the dye solution (concentration: 0.03853 mmol/L) into a thermostated glass vessel (25 °C) containing 0.02 g of the adsorbent. At predetermined time intervals, aliquots were automatically withdrawn from the system and directed through a flow cuvette for UV–Vis spectral acquisition (Cary 100, Varian Inc., Melbourne, VIC, Australia). After each measurement, the sample was returned to the vessel, ensuring a constant solution volume and undisturbed adsorption progress throughout the experiment. During the entire process, the suspension was mechanically stirred at 120 rpm. The changes in absorbance at the wavelength corresponding to the maximum absorption of each dye were converted to concentration changes as a function of time.

## 3. Results and Discussion

### 3.1. Textural and Structural Properties of Mesoporous Carbon Materials

**Figure 1:**
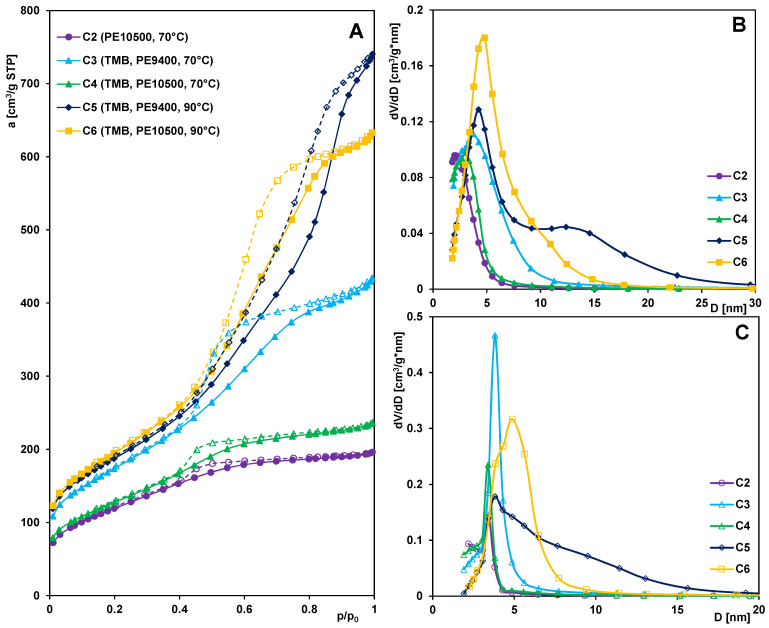
Nitrogen adsorption–desorption isotherms for the mesoporous carbons (**A**). BJH pore size distribution curves from adsorption (**B**) and desorption (**C**) branches of isotherms.

Mesoporous carbon materials were synthesized using the previously obtained silica samples [35] as hard templates and glucose as the carbon precursor. The numerical designations of the carbon samples correspond to silica material precursors. Analysis of the nitrogen adsorption/desorption isotherms (Figure 1A) and the pore size distribution curves obtained from the adsorption and desorption branches (Figure 1B,C) revealed two distinct groups of carbon materials differing in the development of their porous structure—those with relatively medium porosity (carbons C2 and C4) and those with well-developed porous structure (carbons C3, C5, and C6). For the nitrogen adsorption/desorption isotherms of the first group of materials, a relatively small increase in adsorption is observed at low relative pressures, followed by a gradual and systematic rise as the pressure increases. Around a relative pressure of p/p_0_ = 0.4, a hysteresis loop begins to form, which is relatively narrow. The step observed on the adsorption branch, corresponding to capillary condensation in mesopores, is minor. At relative pressures p/p_0_~1, only a slight increase in adsorption is noted. The isotherms of both samples can be classified as type IV with an H1-type hysteresis loop, indicating the presence of pores with shapes close to cylindrical. In the pore size distribution curves derived from the adsorption branch of the isotherms, the peak is incomplete, with its maximum corresponding to pore sizes at the boundary between the micro- and mesopore ranges. The pore size distribution functions obtained from the desorption data exhibit a well-defined, narrow peak in the lower mesopore range, suggesting a relatively high uniformity of the porous structure of the examined materials.

The isotherms of the second group of materials show a similar course up to a relative pressure of approximately p/p_0_ = 0.4, beyond which distinct differences become apparent. For samples C3 and C6, a noticeable step on the adsorption curve extends up to a relative pressure of 0.8, above which the increase in adsorption is relatively small, indicating structural uniformity of the pores. In contrast, for sample C5, the adsorption rise spans a much wider pressure range practically up to p/p_0_ = 1, indicating that this sample possesses a broader pore size distribution. This observation is confirmed by the much wider and less symmetric peaks in the pore size distribution functions (with two well-defined maxima in the PSD derived from the adsorption branch of the isotherm) compared to those of carbons C3 and C6. Carbons C5 and C6 exhibit the most developed porous structure and the highest total pore volume, as evidenced by their significantly higher adsorption values at relative pressures near unity. The isotherms for this group of carbons represent type IV, typical of mesoporous materials, while the hysteresis loops correspond to cylindrical-like pores (type H1 for C3 and C6) and slit-like pores (type H3 for C5).

Table 1 summarizes the textural parameters of the carbon materials together with key data regarding the synthesis conditions and the types of reagents used. As shown by the presented results, certain correlations can be observed between the synthesis procedure and the textural characteristics of the final products. Carbons C2 and C4, obtained by replicating silica templates based on the PE10500 copolymer with or without the use of an expander and aged at 70 °C, are characterized by a less developed specific surface area and a smaller total pore volume (S_BET_ = 431–459 m^2^/g; V_t_ = 0.30–0.37 cm^3^/g). Significantly higher values of these parameters were obtained for carbons C3, C5, and C6 (S_BET_ = 625–698 m^2^/g; V_t_ = 0.67–1.15 cm^3^/g), synthesized from silica materials based on the PE9400 copolymer (regardless of aging temperature) or from PE10500-based silica aged at 90 °C. At the same time, the carbons from the first group exhibit a much smaller average pore diameter (D_BJH ads_ = 3.10–3.42 nm; D_BJH des_ = 3.01–3.29 nm) compared to the other samples (D_BJH ads_ = 4.48–6.92 nm; D_BJH des_ = 4.06–6.36 nm). Regardless of the synthesis procedure used, all carbon materials display a significant contribution of mesopores to the total porosity, ranging from 0.89 to 0.95. Based on the obtained results, it can be concluded that any modification of the synthesis parameters leads to corresponding changes in the textural properties of the resulting material.

The pore size distributions and average pore sizes of carbons were also determined using the KJS method [37], and a comparison of this method and the BJH one is presented in the Supplementary Materials (Figure S1, Table S1). Moreover, a comparison of the texture of carbons and their silica templates [35] was made (Table S2).

**Figure 2:**
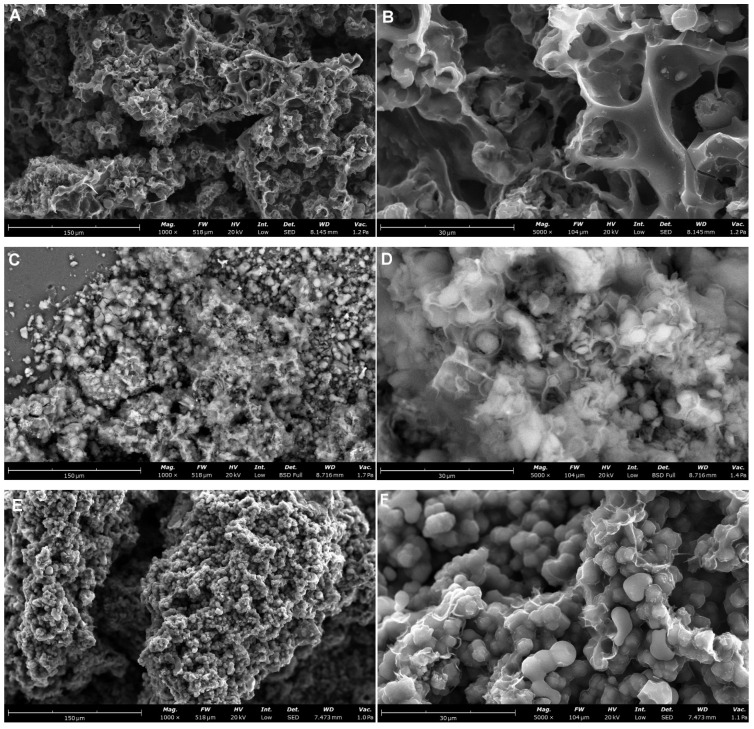

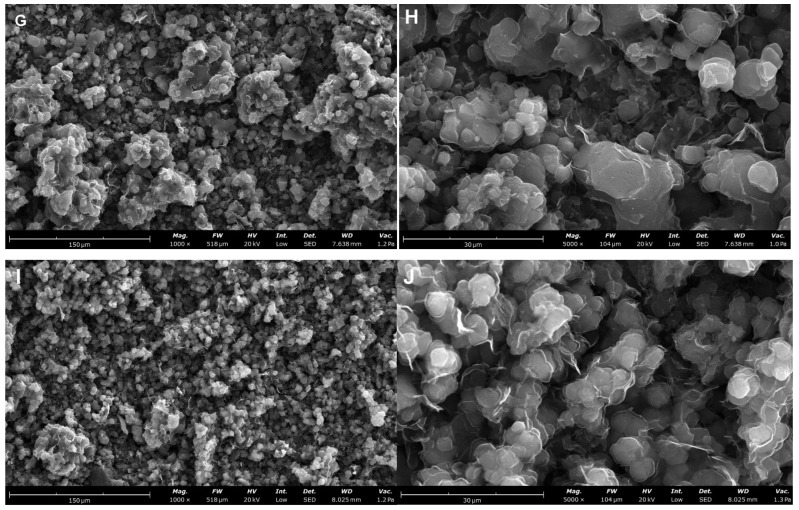
SEM images of the carbon materials C2 (**A**,**B**), C3 (**C**,**D**), C4 (**E**,**F**), C5 (**G**,**H**), and C6 (**I**,**J**) at various magnifications.

The SEM micrographs of the carbon materials (Figure 2) obtained as a replica of the silica template reveal a highly porous structure composed of interconnected carbon particles forming an open, sponge-like framework. At lower magnification (Figure 2A,C,E,G,I), the material exhibits an aggregated morphology with irregularly shaped granules, suggesting that the carbon matrix successfully replicated the porous network of the parent silica structure. The surface appears rough and developed, with numerous interparticle voids that may contribute to the overall mesoporosity of the samples. At higher magnification (Figure 2B,D,F,H,J), the microstructure is characterized by closely packed and partially fused carbon domains, creating curved and convoluted surfaces. The observed morphology indicates the presence of mesopores and macropores formed between the aggregated carbon clusters. The continuity of the porous network and the relatively uniform distribution of the carbon features confirm the effective removal of the silica phase and preservation of the replica’s hierarchical texture.

From the whole group of tested materials, sample C2 shows some morphological differences. The alterations in structural organization observed in the SEM images confirm the structural role of TMB as an effective pore-expanding agent, promoting the generation of a more developed mesoporous network in the carbon replica. SEM images of the C2 sample (Figure 2A,B) obtained without the use of 1,3,5-trimethylbenzene (TMB) as a pore-expanding agent reveals a three-dimensional interconnected pore network with carbon walls forming irregularly shaped voids and channels. The carbon structure without the expander appears denser and less open, with fewer interparticle voids and thicker carbon walls forming a compact framework. The surface exhibits a smoother and more continuous texture, with limited development of interconnected mesopores. In contrast, the carbon replicas prepared using TMB (Figure 2C–J) display a more open and loosely packed architecture composed of smaller carbon domains and abundant interparticle pores, which contribute to its higher specific surface area (from 459 to 698 m^2^/g). The lack of TMB during synthesis likely restricted the formation of larger pore channels, resulting in a reduced BET surface area (431 m^2^/g) and lower textural accessibility.

Transmission electron microscopy (TEM) analyses (Figure S2 in the Supplementary Materials) reveal a well-developed porous carbon structure characterized by a disordered arrangement of pores without long-range periodicity or specific symmetry. The homogeneity at the nanoscale (such as pores uniform in size and shape) was confirmed. The pore walls appear continuous and well-defined.

SAXS (Small-Angle X-ray Scattering) analysis was used to investigate the porous structure of carbon materials, assessing the degree of structure order at the nanometer scale.

**Figure 3:**
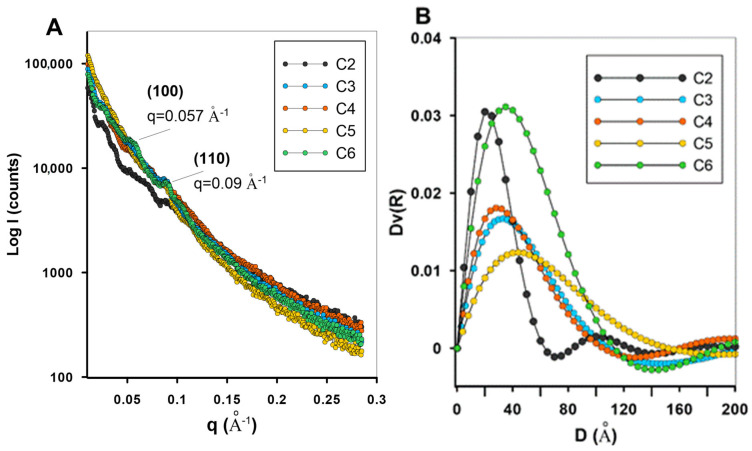
(**A**) The SAXS curves of the investigated carbon materials; (**B**) volumetric distribution of particle or pore radii (Dv(R)).

The SAXS curves recorded for the obtained carbon replicas (Figure 3) exhibit a monotonic decrease in scattering intensity I(q) with increasing scattering vector q, without interference maxima characteristic on a specific mesostructural symmetry. For carbon materials synthesized as replicas of ordered silica templates, the presence of sharp low-angle reflections below q = 1 Å^−1^ corresponding to the at least trace (100) and (110) planes of a hexagonal (p6mm) lattice is indicative of preserved mesoscopic order. In the present carbon samples, the absence of such reflections clearly suggests a loss of long-range structural order during the synthesis process. Only for the C6 sample are some of the curvatures of the scattering curve of such signals observable. In the low region of the scattering curve (below q = 0.05), the SAXS profiles show a smooth decay without great oscillations or peaks related to periodic mesopores. At higher q values (>q = 0.1), corresponding to the Porod region, the intensity follows a power-law dependence which may suggest the presence of surface fractality and rough pore boundaries. Such behavior may indicate lack of specific symmetry but it does not exclude the uniform porosity. The obtained Dv(R) curves (Figure 3B) illustrate the size of the most characteristic scattering elements in the structure of the studied carbons. It should be noted that the dimensions of these objects (maxima on the Dv(R) curve) correspond to the pore sizes obtained by the adsorption method. Furthermore, the obtained Dv(R) signals are quite wide and diffuse, indicating a lack of high order and the presence of a hierarchical pore system.

### 3.2. Surface Chemistry of Mesoporous Carbons

The carbon materials were analyzed by potentiometric titration to determine the chemical nature of their surface. Knowledge of the acid–base properties of such materials is of key importance for adsorption applications, as it allows interpretation of the adsorbent–adsorbate behavior under specific experimental conditions, identification of the type of their mutual interactions, and elucidation of the adsorption mechanism. Based on the potentiometric titration data, the surface charge density of the mesoporous carbons and the position of the point of zero charge (pH_PZC_) were determined (Figure S3 in the Supplementary Materials). The obtained results indicate that the materials exhibit a basic surface character, with the pH_PZC_ values ranging from 8.4 to 10.9. The surface of the carbons is positively charged below the pH_PZC_ and negatively charged at higher pH values. Therefore, it can be concluded that adsorption of non-ionized adsorbate species is expected to proceed efficiently. Electron-rich Lewis basic sites can interact strongly with the π-electrons of aromatic rings present in the adsorbate molecules. In contrast, for ionized adsorbates, electrostatic interactions, either attractive or repulsive interactions are expected to dominate, depending on the ionic form of the adsorbate and the pH of the solution.

**Figure 4:**
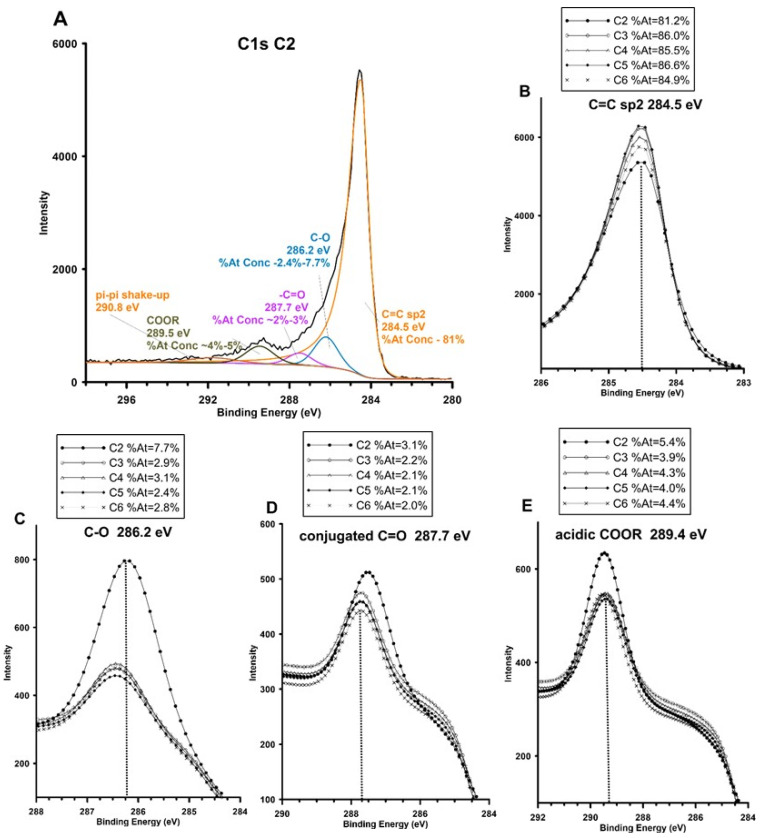
(**A**) High-resolution core-level spectra from the C1s region with identification and contents of individual core levels, and comparison of XPS signals corresponding to individual surface functional groups for the tested C2–C6 carbons: (**B**) C=C sp^2^ groups, (**C**) ether C-O groups, (**D**) conjugated carbonyl and pyrone groups C=O, and (**E**) acidic carboxyl COOR functionalities.

The high pH_PZC_ (>9) of the mesoporous carbons can be rationalized by mapping the basic functional groups by X-ray photoelectron spectroscopy (XPS). The basicity of a carbon surface can arise from the delocalized π electrons within the graphene layers, which can act as Lewis bases [38,39,40] or through certain oxygen-containing groups like chromene, ketone, and pyrone [41,42]. The C1s spectrum of carbon materials C2–C6 is dominated by the sp^2^ carbon peak at ~284.5 eV (Figure 4A,B; ~81–86%), with significant C–O functionalities as ether and surface oxygen sites with free electron pairs (Figure 4C). Ethers and oxidation bridges can act as donor sites and consequently their presence increases the surface basicity (286.0–286.6 eV; ~from 2.4% to 3.1% for C3–C5 and 7.7% for C2 sample, respectively). Conjugated carbonyl and pyrone groups C=O (≈287.7 eV) may exhibit electronic delocalization resulting in π donorship or charge stabilization, which indirectly contributes to the basicity of the carbon surface (Figure 4D). The correctness of the XPS analysis regarding surface basicity was also confirmed based on the analysis of the O1s component. Figure S4 in the Supplementary Materials presents the O1s components most frequently associated with surface basicity, along with their typical binding energies and interpretation. Compared to the observed basic groups, strongly acidic groups remain in the minority (carboxyl component O–C=O (≈289.5 eV; 4–5%) (Figure 4E)). Such a chemical composition implies a reduced, graphitic surface enriched in electron-donating centers (π-planes, ether-like and pyrone/chromene functionalities) and a low density of acidic carboxyl groups, and explains the observed basic surface character and the high affinity toward neutral aromatic adsorbates.

### 3.3. Phase Transformations in Confined Pore Spaces of Mesoporous Carbon Materials

**Figure 5:**
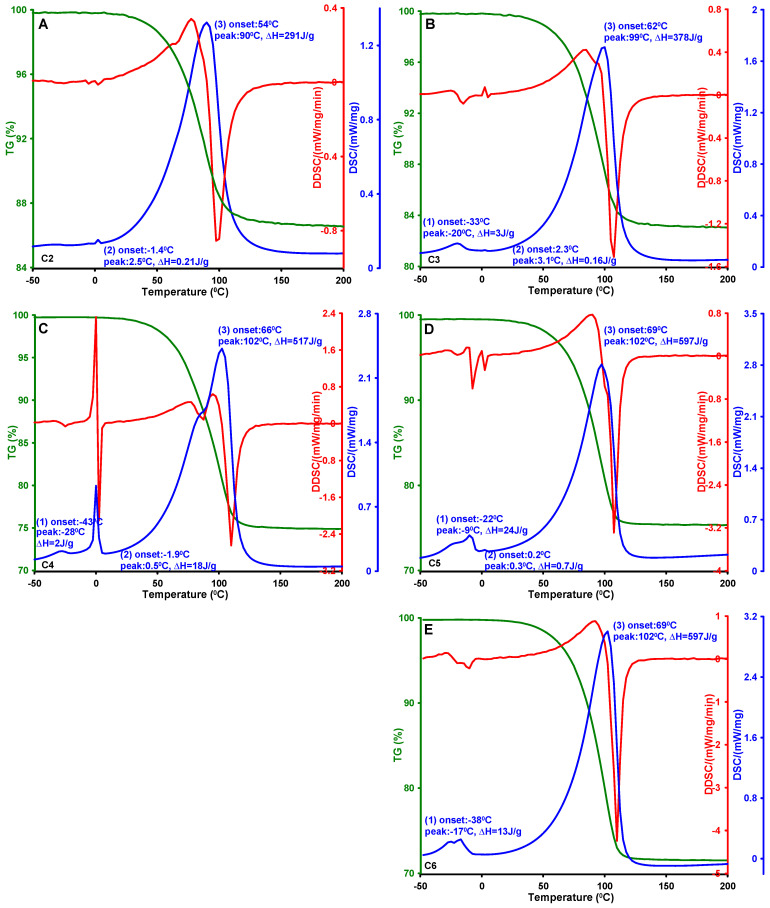
DSC curves for phase transformations occurring in closed pore spaces of mesoporous carbons: C2 (**A**), C3 (**B**), C4 (**C**), C5 (**D**), and C6 (**E**).

Phase transition processes occurring within the spaces of the porous structure were studied in terms of water behavior using the differential scanning calorimetry (DSC) method. Figure 5A–E present the thermograms obtained by differential scanning calorimetry (DSC) for mesoporous carbon samples previously saturated with distilled water. The measurement data are presented in a temperature range from −50 to 200 °C. Three main thermal effects can be distinguished on the recorded DSC curves, corresponding to successive physical transitions: (i) melting of the frozen water confined within the pores; (ii) melting of water frozen on the external surface of the carbon grains or in interparticle spaces; and (iii) evaporation of water from the system into the surroundings.

**Figure 6:**
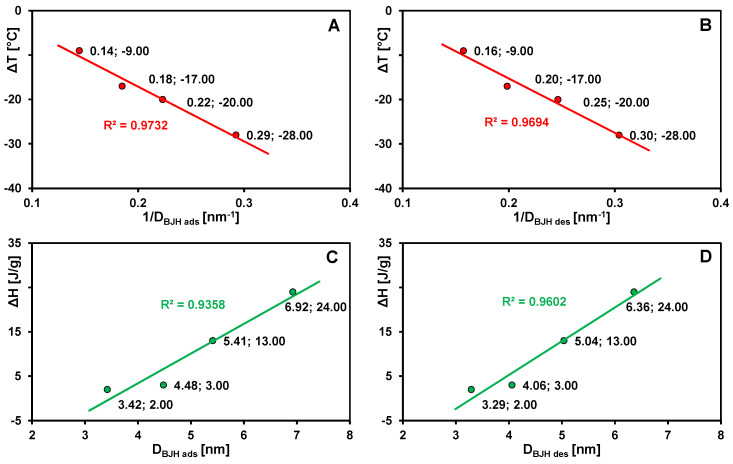
Dependence of the melting point depression (ΔT) on the reciprocal of the average pore radius (1/D_BJH_) determined from the adsorption (**A**) and desorption (**B**) branches of isotherms. Dependence of the specific enthalpy change (ΔH) on the average pore radius (D_BJH_) determined from the adsorption (**C**) and desorption (**D**) branches. ΔT = T_m_ − T_0_, where T_0_ and T_m_ represent the melting temperatures of the bulk liquid and the liquid confined within the pores, respectively.

The first endothermic peak observed on the DSC curves was attributed to the melting of frozen water present within the pores of the analyzed materials. The position of this effect on the temperature axis, as well as the area under the peak, is in strong correlation with the textural properties of the activated carbons. Detailed experimental data, including the onset temperatures (T_on_), the maxima of the endothermic effects (T_max_), and the corresponding enthalpy changes (ΔH), are summarized in Table 2. For sample C2, the signal corresponding to the melting of pore-confined water was very weak and was therefore not included in the analysis. For the remaining samples, a systematic decrease in the characteristic temperatures of pore water melting was observed with decreasing pore diameter, i.e., from −22 to −43 °C for T_on_ and from −9 to −28 °C for T_max_. The dependence of the melting point depression (ΔT) on the reciprocal of the average pore radius (1/D_BJH_), determined from nitrogen adsorption isotherms (Figure 6A,B), is linear (R^2^ = 0.9732 and 0.9694), confirming consistency with the Gibbs–Thomson equation. According to this model, the lowering of the ice melting temperature results from the increased curvature of the water meniscus in narrow pores, which shifts the phase equilibrium in the ice–liquid system. The smaller the pore radius (i.e., the higher the curvature), the lower the melting temperature of the confined water. This phenomenon can also be interpreted in the context of intermolecular interactions. In very narrow pores, water molecules strongly interact with hydrophilic surface groups present on the carbon (carboxyl, carbonyl, and hydroxyl functional groups). These interactions lead to the formation of a tightly bound adsorption layer in which the water loses its translational and rotational mobility, and therefore does not freeze under typical DSC conditions. Only the water molecules located in the central regions of wider pores or in interparticle spaces can form ice structures exhibiting a detectable melting effect.

The dependence of the specific enthalpy change (ΔH) on the average pore radius D_BJH_ (Figure 6C,D) reveals an additional regularity: with increasing pore size (and thus a larger volume of confined water), the contribution of water–carbon surface interactions decreases, while the significance of intermolecular interactions within the water phase increases. This results in an enhancement of the thermal effect associated with the phase transition. The specific enthalpy values, determined from the integrated areas under the DSC peaks, range from 2 to 24 J/g. The analysis of the parameters T_on_ and T_max_, as well as the thermodynamic function ΔH associated with processes (ii) melting of externally frozen water and (iii) evaporation of water, did not show any clear correlations with the porous characteristics of the carbon materials. This indicates that the course of these phase transitions is governed by multiple factors—a complex combination of textural, chemical, and morphological effects. In the case of water evaporation, transport phenomena and vapor pressure differences play a dominant role, making it difficult to establish a simple correlation with pore size.

The correlations of DSC results with the average pore sizes of carbons determined using the KJS method are also presented in the Supplementary Materials (Figure S5).

### 3.4. Adsorption Processes in Porous Carbons

Adsorption techniques play a great role both in technological and environmental applications without introducing expensive and potentially toxic chemical compounds. Apart from industrial adsorption systems or those related to the treatment of water and sewage, a large class of adsorption systems are natural ones—water, sediments, and soil—in which adsorption phenomena determine the retention or migration of pollutants. Understanding the phenomena occurring in such complex systems may only be possible by examining simpler model systems consisting of an adsorbent of defined textural and surface properties and adsorbates of differentiated properties, and by analyzing both the equilibria and kinetics of adsorption processes. Taking into account the effectiveness of adsorption technique, the process should be optimized in order to attain a satisfactory adsorption level and reasonable adsorption rate. Generally, the adsorption effectiveness is determined by adsorbent and adsorbate properties, and conditions in which the process is conducted. The adsorbents should be selected taking into account their affinity towards adsorbed compounds, appropriate pore systems allowing diffusion processes and high adsorption capacities, regeneration ability, and costs. Taking into account the relatively high prices of some adsorbents, there is a need to optimize their use, i.e., to select the appropriate adsorbent and its quantity for the type of adsorption system and specific concentrations or ratios of concentrations of the components of the adsorption system.

**Figure 7:**
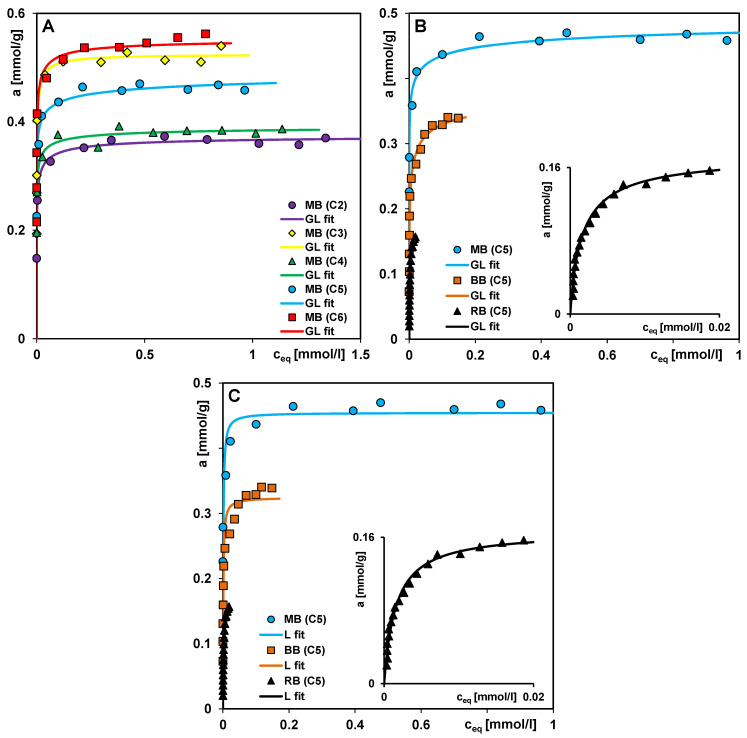
(**A**) Comparison of methylene blue (MB) adsorption isotherms on the mesoporous carbons C2–C6. Comparison of GL (**B**) and L (**C**) isotherms for methylene blue (MB), Bismarck brown (BB), and reactive black (RB) on the mesoporous carbon C5.

The adsorption properties of all investigated mesoporous carbons were evaluated using methylene blue (MB) as a model adsorbate. For the selected carbon (C5), the scope of analysis was extended to include the adsorption of dyes of differentiated structure and properties: Bismarck brown (BB) and reactive black (RB). Figure 7A,B present the adsorption isotherms of methylene blue on the series of studied carbons and the comparison of the adsorption isotherms of the three dyes on carbon C5. Analysis of the methylene blue adsorption isotherms revealed that the adsorption capacity of the carbons decreases in the following order: C6 > C3 > C5 > C4 > C2. The adsorption capacities (a_m_), calculated using the Generalized Langmuir (GL) equation, are 0.55, 0.52, 0.50, 0.39, and 0.38 mmol/g, respectively (Table 3).

**Figure 8:**
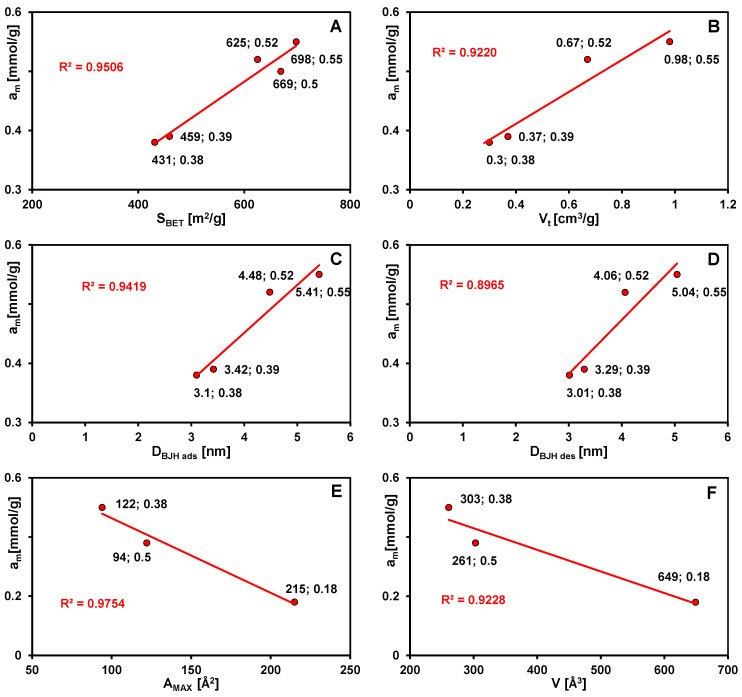
Dependence of the adsorption capacity (a_m_) on the specific surface area (S_BET_) (**A**), the total pore volume (V_t_) (**B**), the average pore radius (D_BJH_) determined from the adsorption (**C**) and desorption (**D**) branches of the mesoporous carbons, dependence of the adsorption capacity (a_m_) on the maximal projection area (A_MAX_) (**E**), and the van der Waals volume of the adsorbate (V) (**F**).

Taking into account the similar surface properties of the synthesized mesoporous carbons, the differences in adsorption capacity can be related mainly to their textural properties, particularly their specific surface area, total pore volume, and average pore radius (Figure 8A–D). In general, an increase in surface area and pore volume leads to improved adsorption performance; however, not all samples follow this trend strictly. Carbon C5, despite exhibiting the highest total pore volume and the broadest pore size distribution, shows a slightly lower adsorption capacity than expected from these correlations. This deviation can be explained by the relationship between the adsorbate molecular size and the pore diameters of the adsorbent. The correlations of adsorption results with the average pore sizes of carbons determined using the KJS method are also presented in the Supplementary Materials (Figure S6).

Methylene blue, being a relatively small and linear molecule, preferentially adsorbs in larger micropores and small mesopores, where surface interactions are stronger. In samples containing a significant proportion of wide mesopores, part of the pore space remains unutilized, resulting in a lower overall adsorption capacity. The equilibrium constant values (log K), obtained from fitting the GL model, range from 1.32 to 1.64 L/mmol (when the assumed range of the optimized parameter was −5 < log K > 3), indicating a good affinity of the mesoporous carbons toward methylene blue. Such relatively high values of this parameter suggest the predominant contribution of dispersive (π–π) interactions occurring between the π-electrons of the aromatic rings of MB molecules and the graphene-like planes of the carbon framework. Additionally, hydrogen bonding and electrostatic interactions may also participate in the adsorption process. In the studied cases, the influence of acidic surface functional groups is limited (acidic surface groups are in the minority as was revealed by XPS analysis). XPS and potentiometric titration indicate the basic nature of the surface (oxygen-containing chromene, ketone, and pyrone groups) and its hydrophobic character (the delocalized π electrons within the graphene layers acting as Lewis bases) [43,44,45], both of which favor the adsorption of aromatic molecules.

Based on adsorption isotherms of the three dyes on carbon C5 (Figure 7B) and their main physicochemical properties (summarized in Table S4), it was observed that the adsorption efficiency decreases in the following order: MB > BB > RB, with the corresponding adsorption capacities (a_m_) of 0.50, 0.38, and 0.18 mmol/g, respectively. The reduction in the amount of dye adsorbed on the carbon material is primarily governed by the increase in molecular size of the respective adsorbates. This relationship is confirmed by the linear correlations between adsorption capacity and the maximal projection area of the adsorbate (A_MAX_), as well as between adsorption capacity (a_m_) and the van der Waals volume of the adsorbate (V) (Figure 8E,F). The highest adsorption efficiency of methylene blue results not only from its smallest molecular dimensions but also from its flat, linear structure, which allows for more compact packing within the porous spaces. In contrast, the BB and RB dyes possess larger and more complex molecules with irregular shapes, which limit their access to smaller pores and may lead to partial blockage of narrow pore channels. Consequently, a portion of the pore volume remains inaccessible to these adsorbates.

Analysis of the equilibrium constant values (log K) revealed that the affinity of carbon C5 toward the studied dyes decreases in the order RB > BB > MB, with the corresponding values of 2.85, 1.63, and 1.32 L/mmol, respectively. The opposite trend compared to the adsorption capacity (a_m_) values indicates that adsorption capacity and adsorption affinity are not directly correlated parameters but arise from different aspects of the adsorption mechanism. The high RB–C5 affinity can be attributed to electrostatic interactions, as under the experimental pH conditions the surface of carbon C5 was positively charged, whereas RB molecules existed in an anionic form. In contrast, methylene blue (MB), being a cationic dye, experienced repulsive electrostatic forces, which reduced its adsorption efficiency despite its smaller molecular size. The obtained results suggest that the adsorption mechanism of the studied dyes on carbon C5 is complex and involves a combination of π–π dispersion interactions, hydrogen bonding, and electrostatic forces. The relative contribution of these interactions depends on the molecular structure of the adsorbate and the surface characteristics of the adsorbent. In processes where steric effects (pore blocking) predominate, the role of electrostatic interactions becomes secondary to diffusion limitations. Reassuming, RB has high affinity (likely due to strong electrostatic forces) but low capacity (due to steric hindrance), while MB has lower affinity (electrostatic repulsion) but can access more pore volume, leading to higher capacity.

As mentioned above, the analysis of the equilibrium data was based on the Generalized Langmuir (GL) equation [46,47,48,49], whose mathematical form is as follows:

(1) $$\theta ={\left[\frac{{\left(Kc_{eq}\right)}^{n}}{1+{\left(Kc_{eq}\right)}^{n}}\right]}^{\frac{m}{n}}$$

where θ = a_eq_/a_m_ is the global adsorption isotherm; a_m_ is the adsorption capacity; m, n are the heterogeneity parameters; and K is the equilibrium constant.

Depending on the specific values of m and n parameters, the GL equation is simplified into four equations: Langmuir (L) (m = n = 1), Langmuir–Freundlich (LF) (0 < m = n < 1), Generalized Freundlich (GF) (n = 1, 0 < m < 1), Tóth (T) (m = 1, 0 < n < 1).

The Generalized Langmuir isotherm (GL) is usually applied for analysis of localized physical adsorption on energetically heterogeneous solids and it describes most of the available experimental data quite well [50,51,52,53]. The experimental systems presented in this work were described using the full form of the GL equation, which assumes adsorption is characterized by an asymmetric quasi-Gaussian energy distribution function, with the heterogeneity parameters m and n in the range 0 < m ≠ n < 1. For the methylene blue and Bismarck brown systems, the fitted values are m = 0.10–0.22 and n = 0.36–0.96, indicating high heterogeneity (lower m and n values correspond to greater heterogeneity). As m < n, these systems display right-extended energy distributions. In contrast, for the reactive black system, both heterogeneity parameters approach unity, reflecting low energetic heterogeneity and, consequently, only slight asymmetry in the energy distribution function. Consequently, this system plus two others were also described using the simplified form of the GL equation with m = n = 1, corresponding to the Langmuir (L) equation (Figure 7C). Although a slight improvement in the fit quality is observed for the RB(C5) system, a pronounced deterioration occurs for the remaining systems (R^2^ = −0.524 and 0.70); in turn, the corresponding adsorption equilibrium constant values appear physically unrealistic. The Langmuir isotherm assumes energetic homogeneity of the adsorption sites. The results obtained for the RB(C5) system can therefore be interpreted as a consequence of a strong match between the size of the adsorbate molecules and the dimensions of the accessible pores. This suggests the existence of a narrow range of pore sizes in which the reactive black molecules can adsorb onto surfaces with similar energetic properties.

A high adsorption capacity enables effective removal of pollution using smaller amounts of material, thereby increasing the cost-effectiveness of the process. Equally important is the adsorption kinetics, particularly under industrial conditions, where the contact time between the liquid and the material is limited. Too slow adsorption may reduce the purification efficiency, even if the adsorbent exhibits a high capacity. To examine this relationship in greater detail, the experimental studies were extended to include measurements of dye adsorption kinetics.

**Figure 9:**
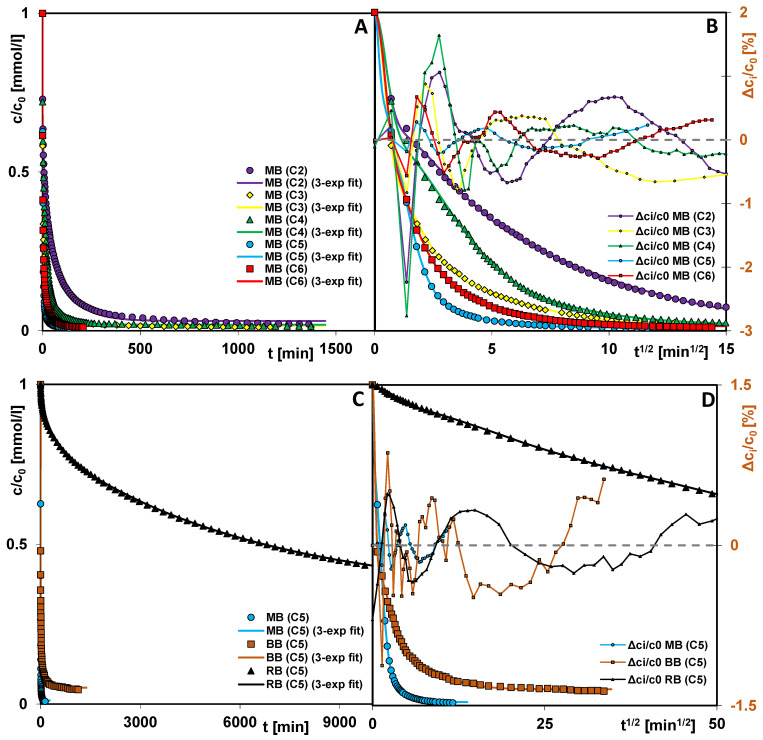
(**A**,**B**) Comparison of adsorption kinetics for methylene blue (MB) on the mesoporous carbons C2–C6. (**C**,**D**) Comparison of adsorption kinetics for methylene blue (MB), Bismarck brown (BB), and reactive black (RB) on the mesoporous carbon C5. Lines correspond to the fitted multi-exponential equation.

Figure 9A–D present the kinetic curves as the dependence of the relative adsorbate concentration on time and on the square root of time. Analysis of the obtained curves indicates that the adsorption behavior of methylene blue is closely correlated with the textural structure of the examined activated carbons. The best adsorption kinetics were observed for carbons C3, C5, and C6, which possess the largest pore diameters, facilitating rapid transport of dye molecules within the internal structure of the solid. For these materials, the values of the t_0.5_ parameter (the time required to reach 50% adsorption relative to the equilibrium amount), determined from a multi-exponential equation, are approximately one minute. The time required to achieve 95% adsorption efficiency (t_95%_) ranges from 15 to 70 min, indicating a highly dynamic process. Particular attention should be paid to sample C5, characterized by the broadest pore size distribution. In this adsorbent, the largest mesopores mainly serve as transport channels, enabling efficient movement of methylene blue molecules deep into the material structure, toward smaller pores where adsorption occurs. This means that not all large pores of this carbon are effectively utilized for adsorption; however, their presence significantly enhances internal diffusion and contributes to the overall high rate of the process. The remaining carbons (C2 and C4), whose pore size distributions are shifted toward smaller diameters, exhibit somewhat slower adsorption kinetics. For these adsorbents, the t_0.5_ values are 7 and 4 min, respectively, while the t_95%_ values reach 290 and 105 min. This indicates that, in their case, the adsorption process is more diffusion-limited, and the transport of dye molecules to active sites occurs with lower efficiency. Despite the noticeable differences in kinetic parameters among the individual samples, methylene blue adsorption on all investigated activated carbons proceeds relatively rapidly. This is due to the dominant contribution of mesopores to the total porosity of the materials (V_p_/V_t_ = 0.89–0.95), which significantly facilitates the diffusion of dye molecules toward the internal adsorption-active surfaces. The obtained results clearly demonstrate that the porous structure of activated carbons, particularly the share and distribution of mesopores, constitutes a key factor determining the adsorption rate and, consequently, the practical efficiency of methylene blue removal from aqueous solutions.

A greater diversity in the adsorption process is observed for systems involving one of three dyes (methylene blue, Bismarck brown, or reactive black) and mesoporous carbon C5. Analysis of the kinetic curves indicates that the highest adsorption rate is exhibited by the system with methylene blue, whereas the slowest with reactive black. For MB, a wide range of linearity is seen in the dependence of concentration on the square root of time, while for RB, linearity is limited to a very narrow range, indicating the presence of transport limitations in the system. In general, as the adsorbate molecule size, molar mass, and structural complexity increase, diffusion both in the solution and within the interfacial region (solution–solid) becomes slower. This phenomenon results from increased diffusion resistance and possible steric effects that restrict the access of molecules to the internal porous surfaces of the adsorbent. It is worth noting, however, that the decrease in adsorption rate for reactive black, compared with the other dyes, is considerably greater than would be expected based solely on differences in structural parameters (2.4–3-fold in molar mass, 1.8–2.3-fold in molecular surface area, and 2.1–2.5-fold in molecular volume). This disproportion may be explained by the structural characteristics of mesoporous carbon C5, which is dominated by slit-shaped pores of irregular geometry. Smaller channels can act as “molecular sieves,” limiting or even completely preventing the diffusion of large molecules into the porous structure. Furthermore, molecules trapped within constrictions may act as steric barriers, restricting the access of other molecules to adsorption-active sites. The described differences in adsorption dynamics among the dyes are clearly reflected in the characteristic times t_95%_. For the systems with methylene blue and Bismarck brown, these times are 39 and 720 min, respectively, whereas for one with reactive black, even after 10,000 min of the experiment, an equilibrium state was not reached, preventing determination of the t_95%_ value. These results indicate that the adsorption mechanism of RB is strongly limited by slow transport in the bulk solution, within the pores, and by probable pore-blocking phenomena.

Additional information on process kinetics is provided by the t_0.5_ parameter values, which are 1.14, 0.37, and 2401 min for MB, BB, and RB adsorption, respectively. The first two values deviate from the general trend observed for the overall process but fall within the range of three initial measurement points. This can be explained by methodological factors, such as potential measurement errors (the rate of dye solution introduction into the adsorbent vessel, delays or premature initiation of spectral recording, variations in particle size distribution, and sample mass differences); and electrostatic factors, arising from the identical charge sign of the C5 carbon surface and the dye molecules. In contrast, under the studied conditions, Bismarck brown exists in a non-ionized form, and therefore its adsorption proceeds without the involvement of electrostatic interactions.

Various equations and kinetic models were applied to analyze the obtained concentration profiles [54,55] (Table S4). The kinetic parameters and statistical quantities used to evaluate the accuracy of individual equations and models are summarized in Table 4. Considering the complexity of the experimental systems (heterogeneity of the porous carbons and the structural complexity of the adsorbate molecules), more advanced semi-empirical equations were selected for kinetic description: the multi-exponential equation (m-exp) and the fractal mixed order equation (f-MOE) [56,57]. The multi-exponential equation (m-exp) describes adsorption as a series of first-order processes occurring simultaneously and/or sequentially. It is commonly employed in the literature to describe processes occurring on energetically or structurally heterogeneous solids that cannot be represented by simple kinetic equations. For the studied dye–mesoporous carbon systems, the equation with three exponential terms was found to provide the optimal fit. As shown in Figure 9A–D, the multi-exponential equation provides a satisfying fit to all experimental systems over nearly the entire measured concentration range. The fractal mixed-order equation (f-MOE) (Figure S7A,B,G,H) introduces a parameter associated with the heterogeneity of the adsorption system—the p fractal factor. For most adsorption systems, p values fall within the range <0, 0.5>, indicating a broad distribution of rate coefficients for individual stages of the adsorption process. For the MB (C5) system, the p value is slightly higher (*p* = 0.59), which correlates well with the shape of its kinetic curve, characterized by a rapid and short adsorption process. In the optimization procedure using the f-MOE equation, the f_2_ parameter generally approached zero, simplifying the equation to the f-FOE form. In terms of fitting quality, the fractal MOE equation yields only slightly less accurate results than the multi-exponential equation.

Among the diffusion-based models, the Crank model (IDM—intraparticle diffusion model) was selected, specifically its variant in which the equilibrium adsorbate uptake (u_eq_) is treated as an additional optimized parameter (Figure S7C,D,I,J). The Crank model assumes that, in the initial stage of the kinetic profile, the experimental data should exhibit linearity in c~t^1/2^ coordinates. However, this behavior was observed only for three systems: MB (C5), MB (C6), and RB (C5). For the remaining systems, large discrepancies between the experimental points and the fitted lines are observed at this stage, as also reflected by the significant deviations of Δc_i_/c_0_ from zero. Moreover, the values of u_eq_ determined for most systems differ substantially from those obtained experimentally. This indicates that the adsorption process is not governed solely by diffusion-related factors (as assumed in the Crank model, where adsorption rate depends only on concentration gradients), but also by other effects such as adsorption interactions and structural and energetic heterogeneity of the adsorbent. As another example of a diffusion-based model, the McKay model (PDM—pore diffusion model) was analyzed (Figure S7E,F,K,L). This model assumes, in addition to diffusion resistance, the presence of an external mass-transfer resistance associated with the passage of adsorbate through a surface boundary layer. As a result, a noticeable improvement in the fitting quality is observed in comparison with the Crank model, particularly in the initial stages of the process. However, the assumption of a constant propagation rate of the adsorbate front at a constant solution concentration leads to the prediction that equilibrium is achieved after a finite time, which contradicts the experimental observations. The inflection points visible in Figure S7L,M correspond to the attainment of equilibrium.

From the above analysis, it appears that the theoretical description of the kinetics of the investigated adsorption systems can be successfully represented using the multi-exponential (m-exp) and fractal mixed order (f-MOE) equations, whereas the diffusion-based models are too simplified and fail to capture the multifactorial nature of the mechanisms governing the observed adsorption processes.

## 4. Conclusions

Mesoporous carbons were obtained as replicas of mesoporous silica materials. Two structural groups were obtained: materials with moderate (C2, C4) and highly developed mesoporosity (C3, C5, C6). Nitrogen adsorption and SEM analyses confirmed effective porous silica network, while SAXS indicated pore systems without long-range order. The pore expander (TMB) proved essential for generating open, accessible architectures.

Potentiometric titration and XPS showed that all carbons possess a strongly basic, graphitic surface enriched in electron-donating oxygen functionalities (pH_PZC_ = 8.4–10.9). Because surface chemistry varied only slightly among the samples, differences in adsorption behavior originate mainly from textural rather than chemical factors.

DSC studies of confined water revealed that melting point depression correlates linearly with the inverse pore diameter, in agreement with the Gibbs–Thomson model. Narrow pores cause stronger water–surface interactions, while larger pores show higher melting enthalpies due to greater contribution of intermolecular interactions within the water phase. Intergrain water melting and water evaporation steps did not correlate clearly with textural parameters.

In adsorption experiments, methylene blue uptake follows the order C6 > C3 > C5 > C4 > C2, governed by surface area, pore volume, and pore size distribution. For carbon C5, adsorption capacity for dyes decreased with molecular size (methylene blue MB > Bismarck brown BB > reactive black RB), confirming steric limitations and partial pore blocking by large molecules. Affinity constants followed the opposite order (RB > BB > MB), highlighting the role of electrostatic interactions.

Kinetic studies showed that carbons with larger mesopores (C3, C5, C6) exhibit the fastest adsorption, with t_0.5_ ~1 min and t_95%_ within 15–70 min for methylene blue. Larger or more complex dye molecules significantly slow diffusion, with t_0.5_ ~2500 min for reactive black. Among the kinetic equations and models evaluated, the multi-exponential and fractal mixed-order equations provided the most accurate description, as they are inherently designed to represent adsorption on solid surfaces exhibiting energetic and/or structural heterogeneity, a category to which the studied carbons belong. This interpretation is supported by SAXS measurements, which indicate a disordered structural framework, and by PSD analysis, which reveals broad pore size distributions. In contrast, the poor performance of simple diffusion models directly reflects the complex, hierarchically porous architecture of these carbon materials.

A comprehensive analysis of the influence of the structural, textural, and surface properties of synthesized carbon materials on the physical processes occurring in closed-pore systems allows for the definition of more general relationships between the parameters characterizing the studied systems and their performance and mechanism. The significant role of porosity is indicated for adsorbates exhibiting diverse molecular sizes, as was the case with the dyes used in this study. It can be argued that it is necessary to design adsorbents or material systems characterized by varying porosity, both to facilitate the transport of adsorbate molecules (large communication pores) and to enhance adsorption forces (smaller pores).

## Figures and Tables

**Table 1:** The textural parameters of carbon materials.

Carbon Sample	C2	C3	C4	C5	C6
Copolymer ^1^ (silica template synthesis)	PE10500	PE9400	PE10500	PE9400	PE10500
Expander ^2^ (silica template synthesis)	-	TMB	TMB	TMB	TMB
T_aging_^3^ [°C]	70	70	70	90	90
S_BET_^4^ [m^2^/g]	431	625	459	669	698
S_ext_ ^5^ [m^2^/g]	4	21	5	15	10
V_t_ ^6^ [cm^3^/g]	0.30	0.67	0.37	1.15	0.98
V_p_ ^7^ [cm^3^/g]	0.29	0.59	0.34	1.07	0.93
V_p_/V_t_ ^8^	0.95	0.89	0.92	0.93	0.95
D_BJH ads_ ^9^ [nm]	3.10	4.48	3.42	6.92	5.41
D_BJH des_ ^10^ [nm]	3.01	4.06	3.29	6.36	5.04

^1^ Pluronic PE-type triblock copolymers as structure-directing agents used for synthesis of mesoporous silica materials with well-organized architectures applied as porous carbon template. ^2^ Expander presence during synthesis of the mesoporous silica as porous carbon template. ^3^ A post-gelation process of silica gel allowing further hydrolysis and condensation of silica phase. ^4^ BET surface area of mesoporous carbons calculated using experimental points at a relative pressure of p/p_0_~0.035–0.31, where p and p_0_ are denoted as the equilibrium and saturation pressure of nitrogen. ^5^ External surface area determined from α_s_ plot. ^6^ Total pore volume calculated by 0.0015468 amount of nitrogen adsorbed at p/p_0_ = 0.99. ^7^ Primary mesopore volume determined from α_s_ plot. ^8^ Mesopore contribution. ^9^ BJH adsorption average pore diameter. ^10^ BJH desorption average pore diameter.

**Table 2:** Characteristic phase transition temperatures in closed pores of carbon materials and the accompanying thermal effects.

Carbon	T_on_ ^1^ [°C]	T_max_ ^1^ [°C]	ΔH ^1^ [J/g]	T_on_ ^2^ [°C]	T_max_ ^2^ [°C]	ΔH ^2^ [J/g]	T_on_ ^3^ [°C]	T_max_ ^3^ [°C]	ΔH ^3^ [J/g]	D_BJH ads_ [nm]	D_BJH des_ [nm]
C2	-	-	-	1.4	2.5	0.21	54	90	291	3.10	3.01
C3	−33	−20	3	2.3	3.1	0.16	62	99	378	4.48	4.06
C4	−43	−28	2	−1.9	0.5	18	66	102	517	3.42	3.29
C5	−22	−9	24	0.2	0.3	0.70	66	97	538	6.92	6.36
C6	−38	−17	13	-	-	-	69	102	597	5.41	5.04

^1^ T—melting temperature of ice in pores. ^2^ T—melting temperature of frozen free water. ^3^ T—evaporation temperature, T_on_—temperature of the onset of the thermal effect, T_max_—maximum peak value, ΔH—specific enthalpy of the process. Due to baseline drift, the onset temperatures (T_on_) may be subject to increased uncertainty.

**Table 3:** Parameters of Generalized Langmuir or Langmuir equations (GL or L) characterizing adsorption of dyes on the mesoporous carbons.

System/Isotherm	a_m_ [mmol/g]	m	n	log K [L/mmol]	R^2^	SD(a)
MB (C2)/GL	0.38	0.15	0.57	1.61	0.992	7.97 × 10^−3^
MB (C3)/GL	0.52	0.12	0.96	1.54	0.979	2.11 × 10^−2^
MB (C4)/GL	0.39	0.12	0.55	1.64	0.975	1.15 × 10^−2^
MB (C5)/GL	0.50	0.10	0.36	1.32	0.982	1.37 × 10^−2^
MB (C5)/L	0.45	1.00	1.00	3.00	−0.524	1.10 × 10^−1^
MB (C6)/GL	0.55	0.10	0.70	1.36	0.911	4.37 × 10^−2^
BB (C5)/GL	0.38	0.22	0.55	1.63	0.988	1.19 × 10^−2^
BB (C5)/L	0.32	1.00	1.00	3.00	0.790	4.43 × 10^−2^
RB (C5)/GL	0.18	0.98	0.87	2.85	0.984	6.21 × 10^−3^
RB (C5)/L	0.17	1.00	1.00	2.81	0.984	5.88 × 10^−3^

**Table 4:** Comparison of kinetic parameters determined based on the various kinetic equations and models.

System	Fit	f_2_/p	log k ^1^	t_0.5_ [min]	u_eq_	SD (c/c_0_) [%]	1 − R^2^
MB (C2)	3-exp	-	−1.02	7.21	0.97	0.66	1.01 × 10^−3^
	f-FOE	0/0.38	−1.30	7.52	1.00	1.18	3.52 × 10^−3^
	IDM	-	−3.77	7.80	0.85	1.86	8.69 × 10^−3^
	PDM	-	-	9.02	0.96	2.81	1.95 × 10^−2^
MB (C3)	3-exp	-	−0.05	0.78	0.98	0.45	6.51 × 10^−4^
	f-FOE	0/0.35	−0.39	0.86	0.99	0.32	3.56 × 10^−4^
	IDM	-	−3.30	0.99	0.91	0.40	5.63 × 10^−4^
	PDM	-	-	0.86	0.99	0.33	3.63 × 10^−4^
MB (C4)	3-exp	-	−0.76	3.96	0.98	0.52	7.47 × 10^−4^
	f-FOE	0/0.47	−0.95	4.12	0.99	1.11	3.73 × 10^−3^
	IDM	-	−2.88	4.11	0.66	1.08	3.48 × 10^−3^
	PDM	-	-	3.90	0.98	0.43	5.38 × 10^−4^
MB (C5)	3-exp	-	−0.22	1.14	0.99	0.15	4.37 × 10^−5^
	f-FOE	0/0.59	−0.27	1.00	0.98	0.71	1.22 × 10^−3^
	IDM	-	−1.76	0.97	0.33	0.97	2.25 × 10^−3^
	PDM	-	-	0.97	0.99	0.95	2.12 × 10^−3^
MB (C6)	3-exp	-	−0.12	0.92	0.99	0.27	2.47 × 10^−4^
	f-FOE	0/0.43	−0.36	0.97	0.99	0.37	4.92 × 10^−4^
	IDM	-	−2.62	0.94	0.79	0.41	6.13 × 10^−4^
	PDM	-	-	0.96	0.99	0.28	2.75 × 10^−4^
BB (C5)	3-exp	-	0.27	0.37	0.95	0.40	5.30 × 10^−4^
	f-MOE	0.29/0.30	−0.63	0.54	0.96	0.98	3.54 × 10^−3^
	IDM	-	−7.10	0.94	0.99	2.02	1.53 × 10^−2^
	PDM	-	-	0.28	0.94	0.74	1.86 × 10^−3^
RB (C5)	3-exp	-	−3.54	2401	0.66	0.23	1.37 × 10^−4^
	f-FOE	0/0.49	−4.17	7039	1.00	0.37	3.85 × 10^−4^
	IDM	-	−6.09	6819	0.66	0.62	1.09 × 10^−3^
	PDM	-	-	6845	0.99	0.35	3.20 × 10^−4^

^1^ k:k_avg_-m-exp (k_avg_ = ln2/t_0.5_); k_1_-f-FOE and f-MOE; B-IDM (B = D/R^2^). t_0.5_:t_0.5avg_-m-exp (t_0.5avg_—overall adsorption half-time determined numerically).

## Data Availability

The original contributions presented in this study are included in the article/Supplementary Material. Further inquiries can be directed to the corresponding authors.

## Supplementary Materials

The following supporting information can be downloaded at: https://www.mdpi.com/article/10.3390/ma19010191/s1, Figure S1: Comparison of the KJS and BJH pore size distributions from adsorption (A.C,E,G,I) of C2–C6, and desorption (B,D,F,H,J) of C2–C6 branches of isotherms.; Figure S2: TEM image of C6 carbon (as example); Figure S3: Dependence of surface charge density on pH for the mesoporous carbons determined by potentiometric titration; Figure S4: High-resolution core-level spectra from the O1s region for C6 carbon (as example) with identification and contents of individual core levels. The O1s components related to the surface basicity are O1s ~530.5–531.8 eV constituting the carbonyl oxygen of Lewis basic character as C=O in ketones and quinones having lone electron pairs capable of accepting a proton or electrostatic interactions, and O1s ~534 eV groups interpreted as ether/bridge oxygen (C–O–C) assigned to ethers, oxygen bridges and epoxy structures. Adsorbed water is not related to alkalinity.; Figure S5: Dependence of the melting point depression (ΔT) on the reciprocal of the average pore radius (1/D_KJS_) determined from the adsorption (A) and desorption (B) branches of isotherms. Dependence of the specific enthalpy change (ΔH) on the average pore radius (D_KJS_) determined from the adsorption (C) and desorption (D) branches. ΔT = T_m_ − T_0_, where T_0_ and T_m_ represent the melting temperatures of the bulk liquid and the liquid confined within the pores, respectively; Figure S6: Dependence of the adsorption capacity (a_m_) on the average pore radius (D_KJS_) determined from the adsorption (A), and desorption (B) branches of the mesoporous carbons; Figure S7: (A–F) Comparison of adsorption kinetics for methylene blue (MB) on the mesoporous carbons C2–C6. (G–L) Comparison of adsorption kinetics for methylene blue (MB), Bismarck brown (BB), and reactive black (RB) on the mesoporous carbon C5. Lines correspond to the fitted: fractal first order equation (f-FOE) (A,B,G,H), Crank model (IDM) (C,D,I,J), McKay model (PDM) (E,F,K,M); Table S1: Comparison of the average pore size of carbons determined by the BJH method with/without KJS correction; Table S2: The textural parameters of silica (S) and carbon (C) materials; Table S3: Physicochemical properties of the dyes; Table S4: Various kinetic equations and models applied to optimize the experimental data.
