# Supplementary material for: Analysis of Physical Processes in Confined Pores of Activated Carbons with Uniform Porosity
Source: Materials (Basel). 2026 Jan 4;19(1):191. doi: 10.3390/ma19010191 (PMC12786414; doi:10.3390/ma19010191)
Supplement: Supplementary file 1 [file materials-19-00191-s001.zip › materials-4026459-supplementary.pdf]

Analysis of Physical Processes in Confined Pores of Activated Carbons with Uniform Porosity

Magdalena Blachnio [1,*], Malgorzata Zienkiewicz-Strzalka [1] and Anna Derylo-Marczewska [1,*]

[1] Department of Physical Chemistry, Institute of Chemical Sciences, Maria Curie-Sklodowska University, Maria Curie-Sklodowska Square 3, 20-031 Lublin, Poland; malgorzata.zienkiewicz-strzalka@mail.umcs.pl (M.Z.-S.)

* Correspondence: anna.derylo-marczewska@mail.umcs.pl (A.D.-M.); magdalena.blachnio@mail.umcs.pl (MB); Tel.: +48-0815375637

Introducing the Kruk-Jaroniec-Sayari correction to the BJH method for determination of the adsorption/desorption pore distributions for carbon materials (Figure S1) shifts the curves toward larger pore diameters, while their shapes, although similar, are more irregular. Both bimodal and heterogeneous distributions can be distinguished. Consequently, an increase in the average pore size is also noted (Table S1). Despite these changes in textural data, the correlations of DSC/adsorption results with the average pore size of the samples remained (Figures S5 and S6).

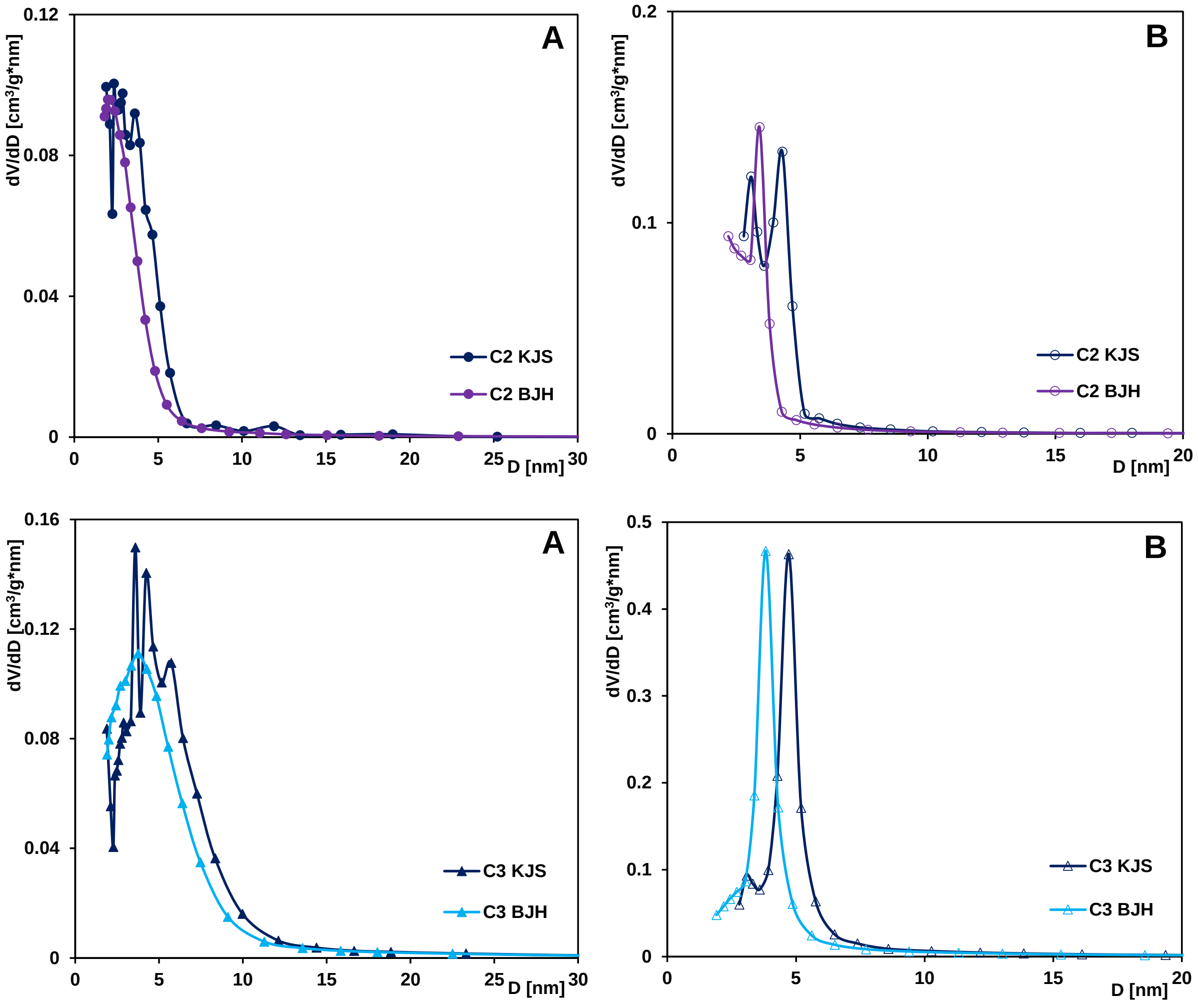

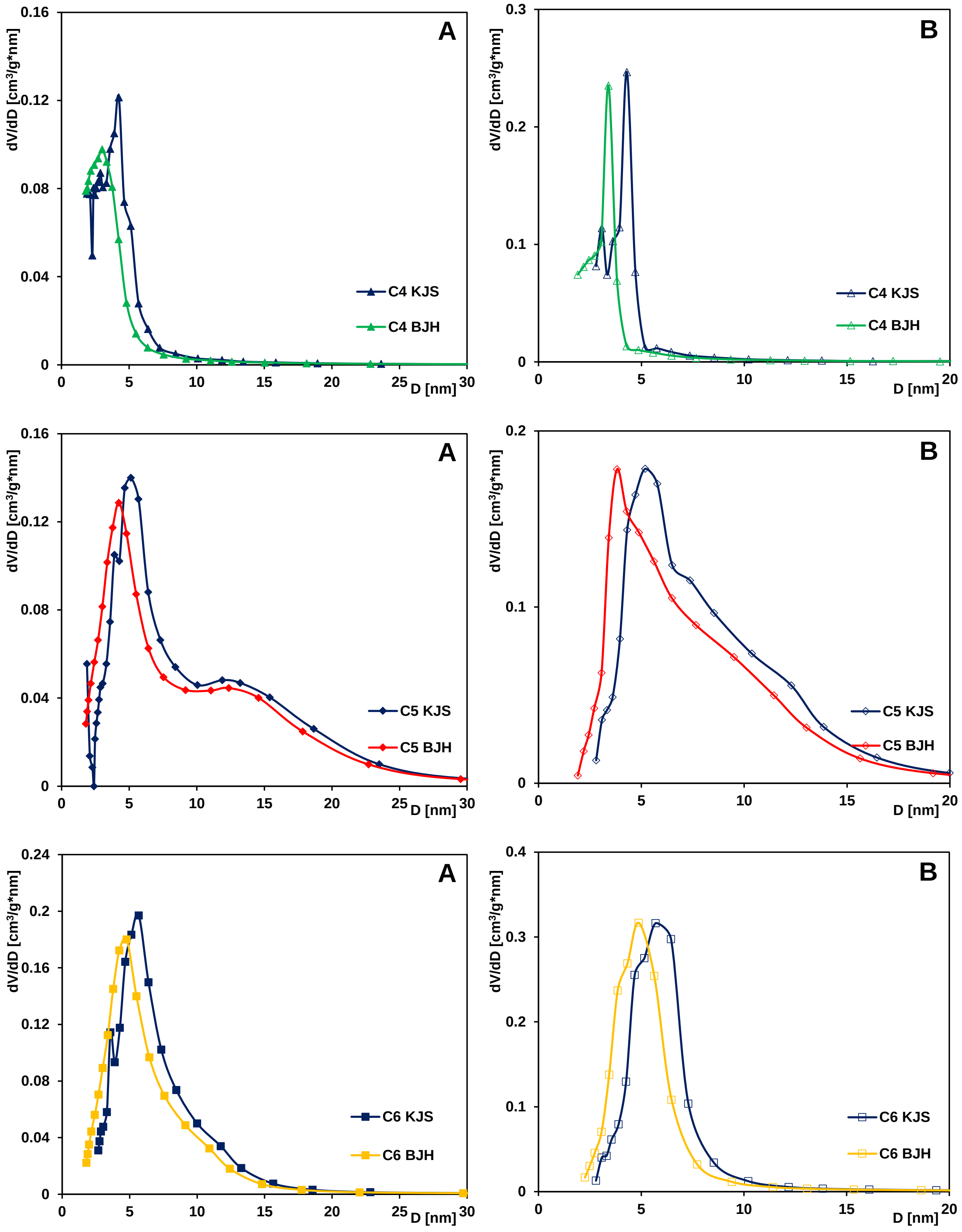

Figure S1. Comparison of the KJS and BJH pore size distributions from adsorption (**A**), and desorption (**B**) branches of isotherms.

Table S1. Comparison of the average pore size of carbons determined by the BJH method with/without KJS correction.

Sample	C2	C3	C4	C5	C6
$D_{KJS\ ads}/D_{BJH\ ads}$[1] [nm]	3.43/3.10	4.98/4.48	3.83/3.42	7.67/6.92	6.16/5.41
$D_{KJS\ des}/D_{BJH\ des}$[2] [nm]	3.95/3.01	5.01/4.06	4.28/3.29	7.32/6.36	5.86/5.04

[1]BJH adsorption average pore diameter with/without KJS correction. [2] BJH desorption average pore diameter with/without KJS correction

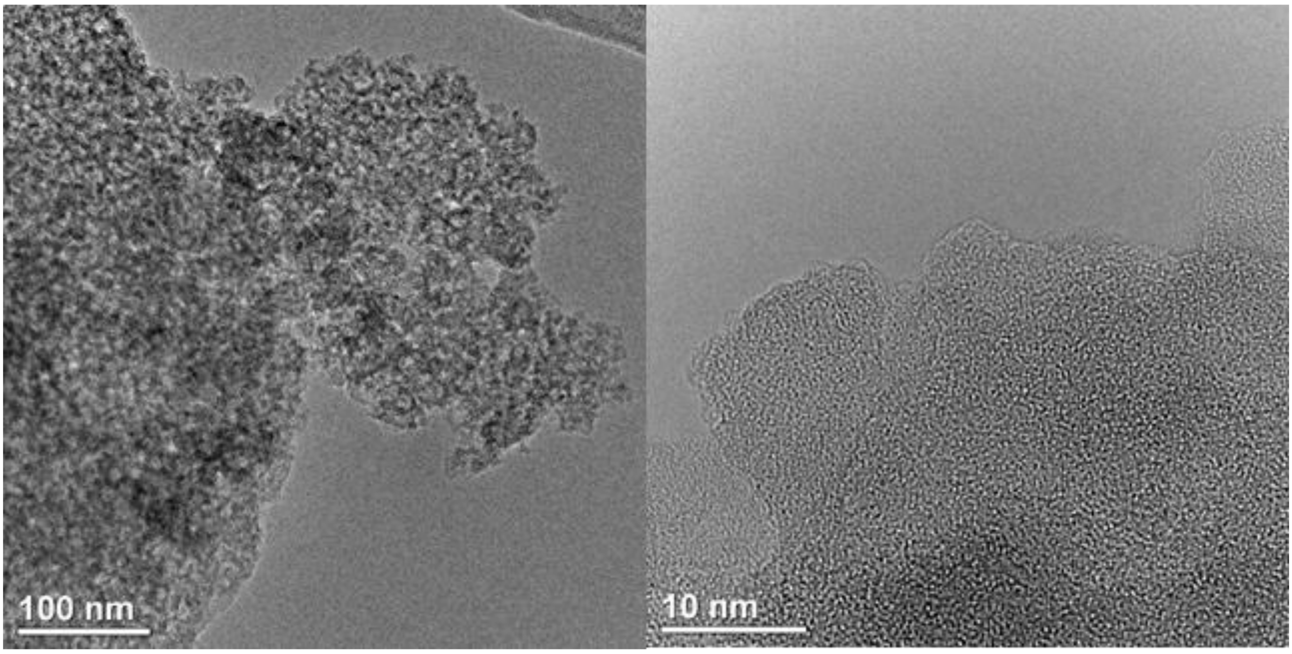

Figure S2. TEM image of C6 carbon (as example).

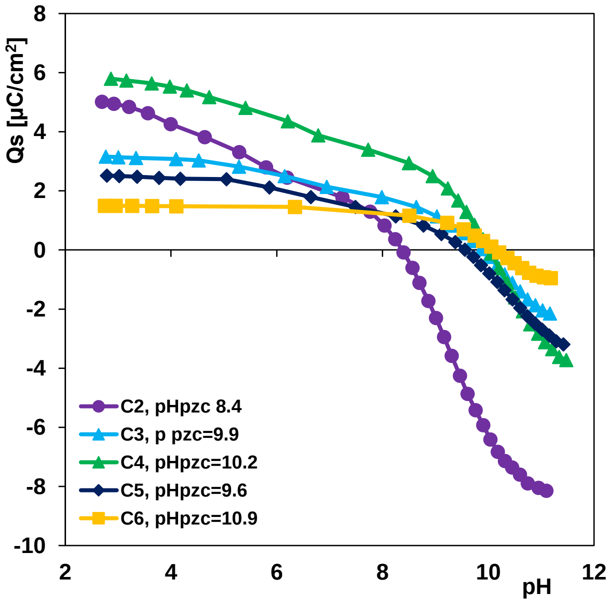

Figure S3. Dependence of surface charge density on pH for the mesoporous carbons determined by potentiometric titration.

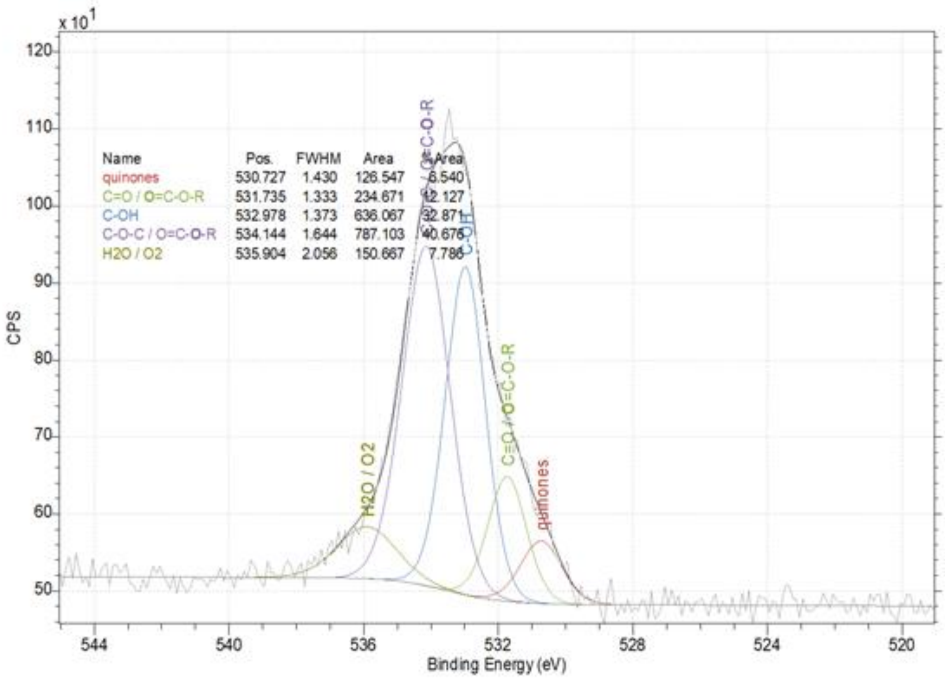

Figure S4. High-resolution core-level spectra from the O1s region for C6 carbon (as example) with identification and contents of individual core levels. The O1s components related to the surface basicity are O1s ~530.5–531.8 eV constituting the carbonyl oxygen of Lewis basic character as C=O in ketones and quinones having lone electron pairs capable of accepting a proton or electrostatic interactions, and O1s ~534 eV groups interpreted as ether/bridge oxygen (C–O–C) assigned to ethers, oxygen bridges and epoxy structures. Adsorbed water is not related to alkalinity.

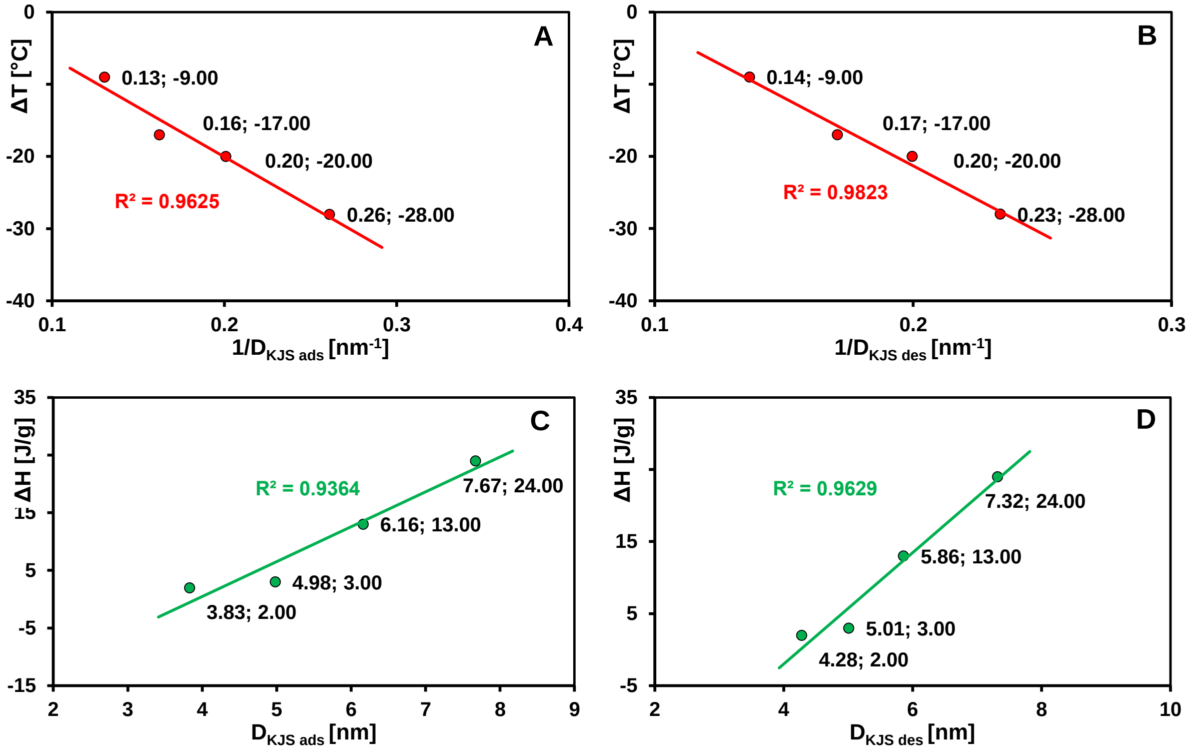

Figure S5. Dependence of the melting point depression (ΔT) on the reciprocal of the average pore radius ($1/D_{KJS}$) determined from the adsorption (**A**) and desorption (**B**) branches of isotherms. Dependence of the specific enthalpy change (ΔH) on the average pore radius (D_{KJS}) determined from the adsorption (**C**) and desorption (**D**) branches. $\Delta T = T_m - T_0$, where T_0 and T_m represent the melting temperatures of the bulk liquid and the liquid confined within the pores, respectively.

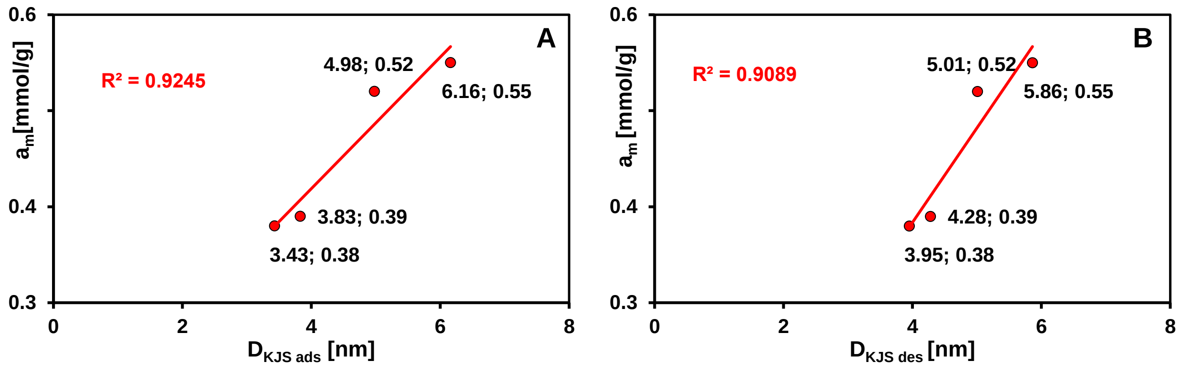

Figure S6. Dependence of the adsorption capacity (a_m) on the average pore radius (D_{KJS}) determined from the adsorption (**A**), and desorption (B) branches of the mesoporous carbons.

The obtained carbon materials, synthesized using silica-based templates exhibit lower developed surface areas and total pore volumes compared to the initial materials (S_{BET}: 431-698 vs. 689-944 m²/g and V_t: 0.30-1.15 vs. 0.50-1.29 cm³/g) (Table 2S). For most samples, a change in pores shape is observed, indicating an inaccurate replication of the used templates. A transformation from bottle-shaped pores (H2), characterized by narrow neck and wide cell, to cylindrical (H1) or slit-like (H3) pores is observed. Additionally, a reduction in average pore diameters is also noted, especially for samples labelled 3–6 determined from the adsorption branches of isotherms ($D_{BJH\ ads}$: 5.39-8.09 vs. 3.42-6.92 nm). Despite these textural changes resulting from various factors in the subsequent stages of template impregnation with the carbon precursor and silica etching, the contribution of mesopores in the total porosity of carbon materials remains relatively high (V_p/V_t:0.90–0.95), indicating a uniform porosity.

Table S2. The textural parameters of silica (S) and carbon (C) materials.

Sample	S2/C2	S3/C3	S4/C4	S5/C5	S6/C6
S_{BET}[1] [m²/g]	759/431	792/625	689/459	852/669	944/698
V_t[2] [cm³/g]	0.50/0.30	0.91/0.67	0.66/0.37	1.29/1.15	1.08/0.98
V_p[3] [cm³/g]	0.49/0.29	0.87/0.59	0.63/0.34	1.18/1.07	1.03/0.93
V_p/V_t[4]	0.98/0.95	0.96/0.89	0.95/0.92	0.91/0.93	0.96/0.95
$D_{BJH\ ads}$[5] [nm]	3.25/3.10	6.70/4.48	5.39/3.42	8.09/6.92	6.01/5.41
$D_{BJH\ des}$[6] [nm]	3.08/3.01	3.95/4.06	3.92/3.29	4.20/6.36	4.00/5.04

[1]BET surface area of solids calculated using experimental points at a relative pressure of p/p_0~ 0.035-0.31, where, p and p_0 are denoted as the equilibrium and saturation pressure of nitrogen. [2]Total pore volume calculated by 0.0015468 amount of nitrogen adsorbed at p/p_0=0.99. [3]Primary mesopore volume determined from α_s plot. [4]Mesopore contribution. [5]BJH adsorption average pore diameter. [6] BJH desorption average pore diameter.

Table S3. Physicochemical properties of the dyes.

Dye code	Chemical formula	Molecular weight [g/mol]	Ionization constant, pK_a	Maximal projection area, A_{max} [Å²]	Van der Waals volume, V [Å³]
MB	$C_{16}H_{18}N_3ClS$	319.85	2.6; 11.2	94	261
BB	$C_{18}H_{18}N_8$ 2HCl	419.31	5.0	122	303
RB	$C_{26}H_{21}N_5Na_4O_{19}S_6$	991.82	3.8; 6.9	215	649

Table S4. Various kinetic equations and models applied to optimize the experimental data.

Kinetic equation	Mathematical form
Multi-exponential equation (m-exp)	$c = (c_o - c_{eq})\sum_{i=1}^{n} f_i exp(-k_i t) + c_{eq}$ (1)<br>or $c = c_0 - c_0 u_{eq} \sum_{i=1}^{n} f_i[1 - exp(-k_i t)]$ (2)<br>where "i" is the term of m-exp equation, k_i is the rate coefficient and $u_{eq}=1-c_{eq}/c_o$ is the relative loss of adsorbate from the solution.
Fractal-like MOE equation (f-MOE)	$F = \frac{1 - exp(-k_1 t)^p}{1 - f_2 exp(-k_1 t)^p}$ (3)
Fractal-like FOE equation (f-FOE)	$F = 1 - exp(-k_1 t)^p$ (4)<br>where p is the fractal coefficient.
Intraparticle Diffusion Model (IDM, Crank)	$F = 1 - \frac{6}{\pi^2}\sum_{n=1}^{\infty}\frac{1}{n^2} exp\left(\frac{-\pi^2 \cdot n^2 \cdot D_a \cdot t}{r^2}\right)$ (5)<br>where r is the radius of the adsorbent particle, D_a is the effective diffusion coefficient:<br>$D_a = \frac{D}{\tau_p \cdot (1 + \rho \cdot K_H \cdot \varepsilon_p)}$ (6)<br>where D is the molecular diffusion coefficient, τ_p – is the dimensionless pore tortuosity factor, ρ is the particle density, ε_p is the particle porosity, K_H – is the Henry adsorption constant.
Pore Diffusion Model (PDM, McKay)	$\frac{dF}{d\tau_s} = \frac{3(1 - u_{eq} \cdot F) \cdot (1 - F)^{\frac{1}{3}}}{1 - B \cdot (1 - F)^{\frac{1}{3}}}$ (7)<br>where u_{eq} is the relative adsorbate loss, the parameter $B = 1\text{-}1/B_i$, *where* $B_i = K_f/D_p$ is the Biot number, D_p is the pore diffusion coefficient, K_f is the external mass transfer coefficient, τ_s is the undersized model time:<br>$\tau_s = \frac{1}{6 \cdot u_{eq}}\left\{\left(2B - \frac{1}{b}\right) \cdot ln\left[\left\|\frac{x^3 + X^3}{1 + X^3}\right\|\right] + \frac{3}{a} ln\left[\left\|\frac{x + X}{1 + X}\right\|\right]\right\} + \left\{arc\tan\left(\frac{2 - X}{X \cdot \sqrt{3}}\right)\right\} - arc\tan\left(\frac{2 \cdot x - X}{X \cdot \sqrt{3}}\right)$ (8)<br>where: $x = (1 - F)^{\frac{1}{3}}, b = \left(\frac{1}{1 - u_{eq}}\right)^{\frac{1}{3}}$ (9)

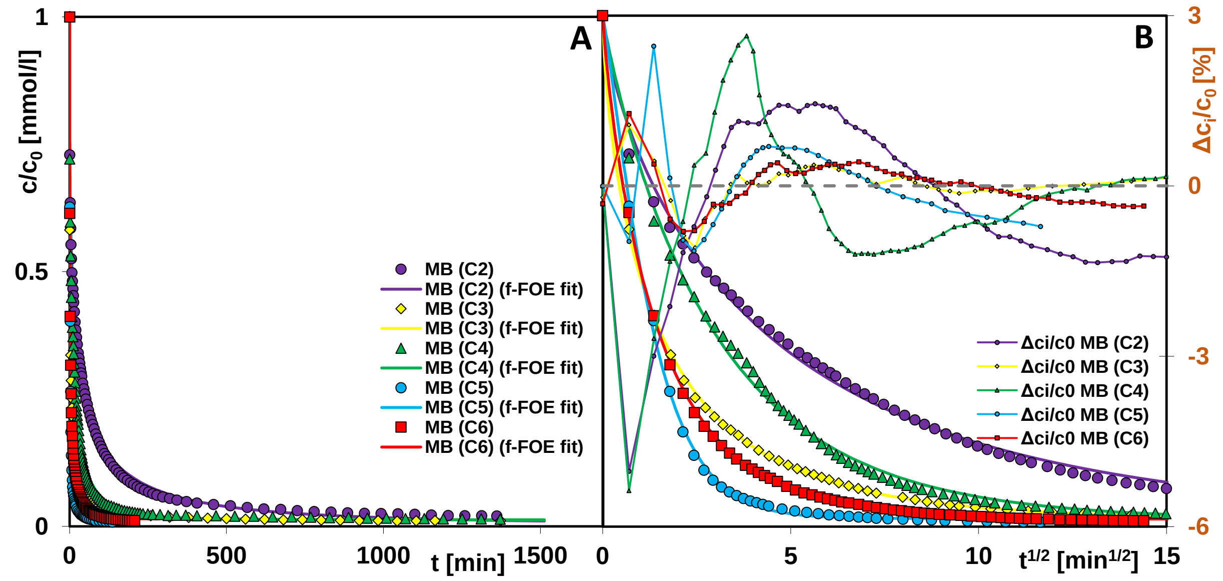

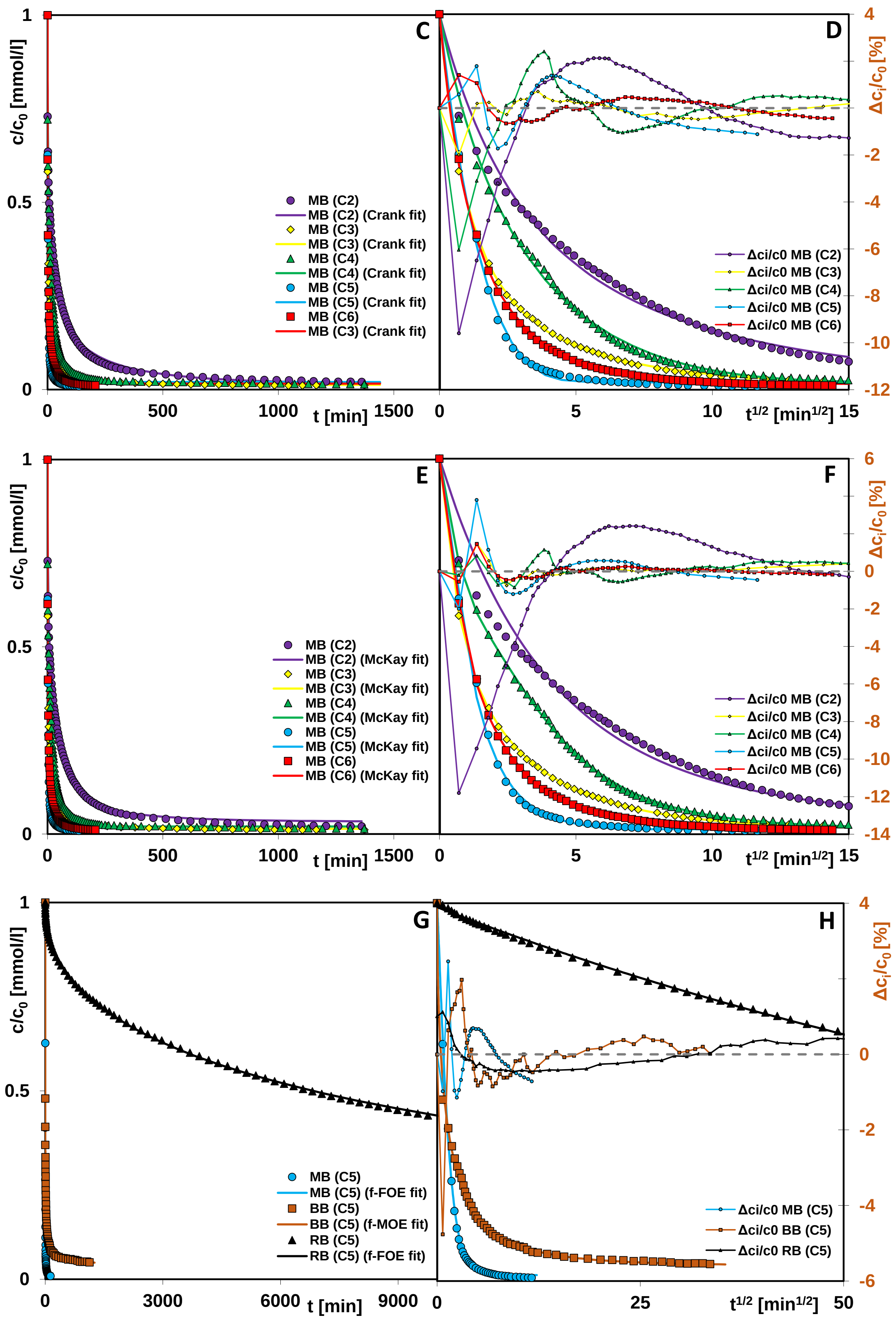

C
D
E
F
G
H
c/c0 [mmol/l]
t [min]
t^1/2 [min^1/2]
Δci/c0 [%]
MB (C2)
MB (C2) (Crank fit)
MB (C3)
MB (C3) (Crank fit)
MB (C4)
MB (C4) (Crank fit)
MB (C5)
MB (C5) (Crank fit)
MB (C6)
MB (C3) (Crank fit)
Δci/c0 MB (C2)
Δci/c0 MB (C3)
Δci/c0 MB (C4)
Δci/c0 MB (C5)
Δci/c0 MB (C6)
MB (C2) (McKay fit)
MB (C3) (McKay fit)
MB (C4) (McKay fit)
MB (C5) (McKay fit)
MB (C6) (McKay fit)
MB (C5) (f-FOE fit)
BB (C5)
BB (C5) (f-MOE fit)
RB (C5)
RB (C5) (f-FOE fit)
Δci/c0 BB (C5)
Δci/c0 RB (C5)

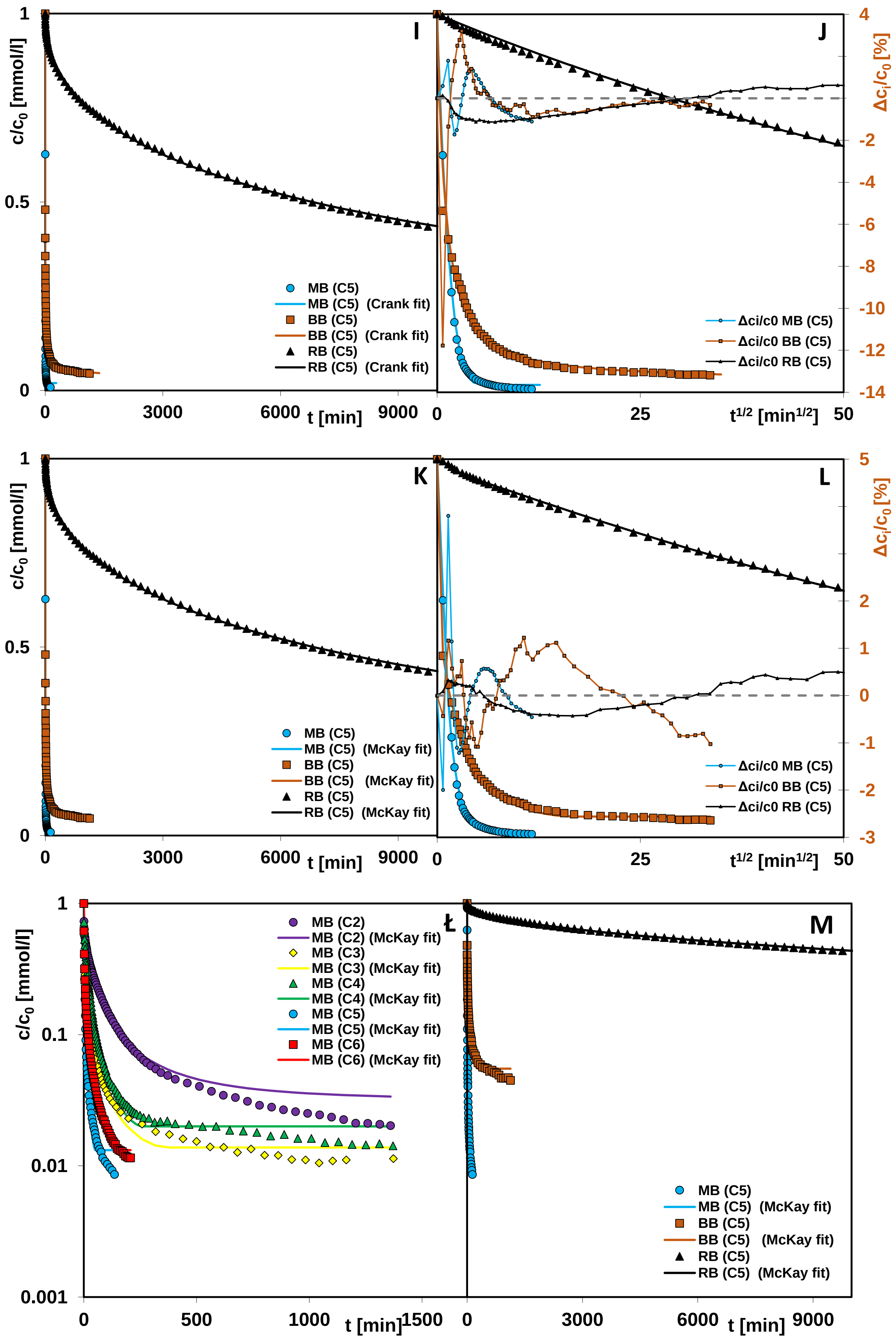

I
J
K
L
Ł
M
c/c0 [mmol/l]
t [min]
t1/2 [min1/2]
Δci/c0 [%]
MB (C5)
MB (C5) (Crank fit)
BB (C5)
BB (C5) (Crank fit)
RB (C5)
RB (C5) (Crank fit)
Δci/c0 MB (C5)
Δci/c0 BB (C5)
Δci/c0 RB (C5)
MB (C5) (McKay fit)
BB (C5) (McKay fit)
RB (C5) (McKay fit)
MB (C2)
MB (C2) (McKay fit)
MB (C3)
MB (C3) (McKay fit)
MB (C4)
MB (C4) (McKay fit)
MB (C6)
MB (C6) (McKay fit)

Figure S7. (**A-F**) Comparison of adsorption kinetics for methylene blue (MB) on the mesoporous carbons C2-C6. (**G-L**) Comparison of adsorption kinetics for methylene blue (MB), Bismarck brown (BB), and reactive black (RB) on the mesoporous carbon C5. Lines correspond to the fitted : fractal first order equation (f-FOE) (**A, B, G, H**), Crank model (IDM) (**C, D, I, J**), McKay model (PDM) (**E, F, K-M**).
